# Supplementary material for: Chemical reversible crosslinking enables measurement of RNA 3D distances and alternative conformations in cells
Source: Nat Commun. 2022 Feb 17;13:911. doi: 10.1038/s41467-022-28602-3 (PMC8854666; doi:10.1038/s41467-022-28602-3)
Supplement: Supplementary file 1 — Supplementary Information [file 41467_2022_28602_MOESM1_ESM.pdf]

SUPPLEMENTARY INFORMATION

Chemical Reversible Crosslinking Enables Measurement of RNA 3D Distances and Alternative Conformations in Cells

Ryan Van Damme,[1,§] Kongpan Li,[1,§] Minjie Zhang,[1,§] Jianhui Bai,[1] Wilson H. Lee,[1] Joseph D. Yesselman,[2] Zhipeng Lu [1*] and Willem A. Velema [3*]

[1]Department of Pharmacology and Pharmaceutical Sciences, School of Pharmacy, University of Southern California, Los Angeles, CA 90033

[2]Department of Chemistry, University of Nebraska-Lincoln, 832A Hamilton Hall, Lincoln, NE, 68588, USA

[3]Institute for Molecules and Materials, Radboud University Nijmegen, The Netherlands

[§]These authors contributed equally to this work.

To whom correspondence should be addressed: zhipengl@usc.edu, willem.velema@ru.nl

Characterization of activated dicarboxylic acids.

Oxalic acid imidazolide: *1,2-di(1H-imidazol-1-yl)ethane-1,2-dione.* ^{1}H NMR (400 MHz, DMSO) δ 8.23 (d, *J* = 1.1 Hz, 1H), 7.58 (t, *J* = 1.4 Hz, 1H), 7.05 – 7.00 (m, 1H). ^{13}C NMR (101 MHz, DMSO) δ 162.39, 138.16, 130.47, 116.87. ESI-MS: [M+H] calculated: 191.1 ; Found: 191.8

Succinic acid imidazolide: *1,4-di(1H-imidazol-1-yl)butane-1,4-dione.* ^{1}H NMR (400 MHz, DMSO) δ 8.52 (t, *J* = 1.1 Hz, 2H), 7.77 (t, *J* = 1.5 Hz, 2H), 7.12 (dd, *J* = 1.7, 0.8 Hz, 2H), 3.49 (s, 4H). ^{13}C NMR (101 MHz, DMSO) δ 169.98, 137.61, 130.91, 116.99, 29.65. ESI-MS: [M+H] calculated: 219.1 ; Found: 219.2

Glutaric acid imidazolide: *1,5-di(1H-imidazol-1-yl)pentane-1,5-dione.* ^{1}H NMR (400 MHz, DMSO) δ 8.42 (t, *J* = 1.1 Hz, 2H), 7.71 (t, *J* = 1.5 Hz, 2H), 7.08 (dd, *J* = 1.7, 0.8 Hz, 2H), 3.16 (t, *J* = 7.0 Hz, 4H), 2.06 (p, *J* = 7.0 Hz, 2H). ^{13}C NMR (101 MHz, DMSO) δ 170.58, 137.39, 130.69, 116.91, 33.80, 18.27. ESI-MS: [M+H] calculated: 233.1 ; Found: 233.2

Diglycolic acid imidazolide: *2,2'-oxybis(1-(1H-imidazol-1-yl)ethan-1-one).* ^{1}H NMR (400 MHz, DMSO) δ 8.41 (t, *J* = 1.1 Hz, 2H), 7.71 (t, *J* = 1.5 Hz, 2H), 7.10 (dd, *J* = 1.7, 0.8 Hz, 2H), 5.04 (s, 4H). ^{13}C NMR (101 MHz, DMSO) δ 167.93, 137.10, 130.66, 116.55, 69.65. ESI-MS: [M+H] calculated: 235.1 ; Found: 235.1

Terephthalic acid imidazolide: *1,4-phenylenebis((1H-imidazol-1-yl)methanone).* ^{1}H NMR (400 MHz, DMSO) δ 8.28 (t, *J* = 1.1 Hz, 2H), 8.02 (s, 4H), 7.76 (t, *J* = 1.5 Hz, 2H), 7.25 – 7.16 (m, 2H). ^{13}C NMR (101 MHz, DMSO) δ 165.97, 139.25, 136.03, 131.20, 130.37, 118.73. ESI-MS: [M+H] calculated: 267.1 ; Found: 267.0

Isocinchomeronic acid imidazolide: *pyridine-2,5-diylbis((1H-imidazol-1-yl)methanone).* ^{1}H NMR (400 MHz, DMSO) δ 9.16 (dd, *J* = 2.2, 0.8 Hz, 1H), 8.71 (t, *J* = 1.0 Hz, 1H), 8.54 (dd, *J* = 8.1, 2.2 Hz, 1H), 8.37 – 8.31 (m, 2H), 7.97 (dd, *J* = 1.7, 1.2 Hz, 1H), 7.81 (t, *J* = 1.5 Hz, 1H), 7.23 (dd, *J* = 1.7, 0.8 Hz, 1H), 7.19 (dd, *J* = 1.7, 0.8 Hz, 1H). ^{13}C NMR (101 MHz, DMSO) δ 164.53, 163.36, 152.20, 149.66, 140.30, 139.77, 139.32, 131.74, 131.38, 130.69, 126.34, 124.68, 118.68, 118.61. ESI-MS: [M+H] calculated: 268.1 ; Found: 268.2

6,6'-binicotinic acid imidazolide: *[2,2'-bipyridine]-5,5'-diylbis((1H-imidazol-1-yl)methanone).* ^{1}H NMR (400 MHz, DMSO) δ 9.16 (dd, *J* = 2.2, 0.8 Hz, 2H), 8.69 (dd, *J* = 8.2, 0.9 Hz, 2H), 8.46 (dd, *J* = 8.3, 2.3 Hz, 2H), 8.35 (d, *J* = 1.1 Hz, 2H), 7.81 (t, *J* = 1.5 Hz, 2H), 7.22 (d, *J* = 1.6 Hz, 2H). ^{13}C NMR (101 MHz, DMSO) δ 167.89, 157.57, 150.81, 139.70, 139.09, 131.21, 129.46, 121.58, 118.77. ESI-MS: [M+H] calculated: 345.1 ; Found: 345.2

Dipicolinic acid imidazolide (DPI for short): *pyridine-2,6-diylbis((1H-imidazol-1-yl)methanone)*. ^{1}H NMR (400 MHz, dmso) δ 8.67 (dd, *J* = 1.3, 0.8 Hz, 2H), 8.51 – 8.37 (m, 3H), 7.94 (dd, *J* = 1.7, 1.2 Hz, 2H), 7.18 – 7.11 (m, 2H). ^{13}C NMR (101 MHz, DMSO) δ 163.1, 148.8, 140.5, 140.1, 130.6, 130.2, 118.8. ESI-MS: [M+H] calculated: 268.1 ; Found: 268.3.

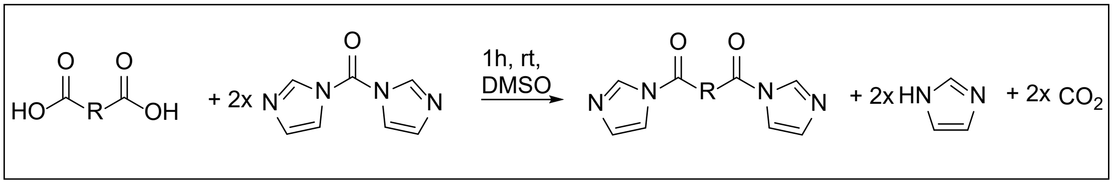

Synthesis of crosslinkers from dicarboxylic acids and CDI.

Supplementary Figure 1. Synthetic Scheme, Mass spectrometry and NMR characterization of SHARC reagents.

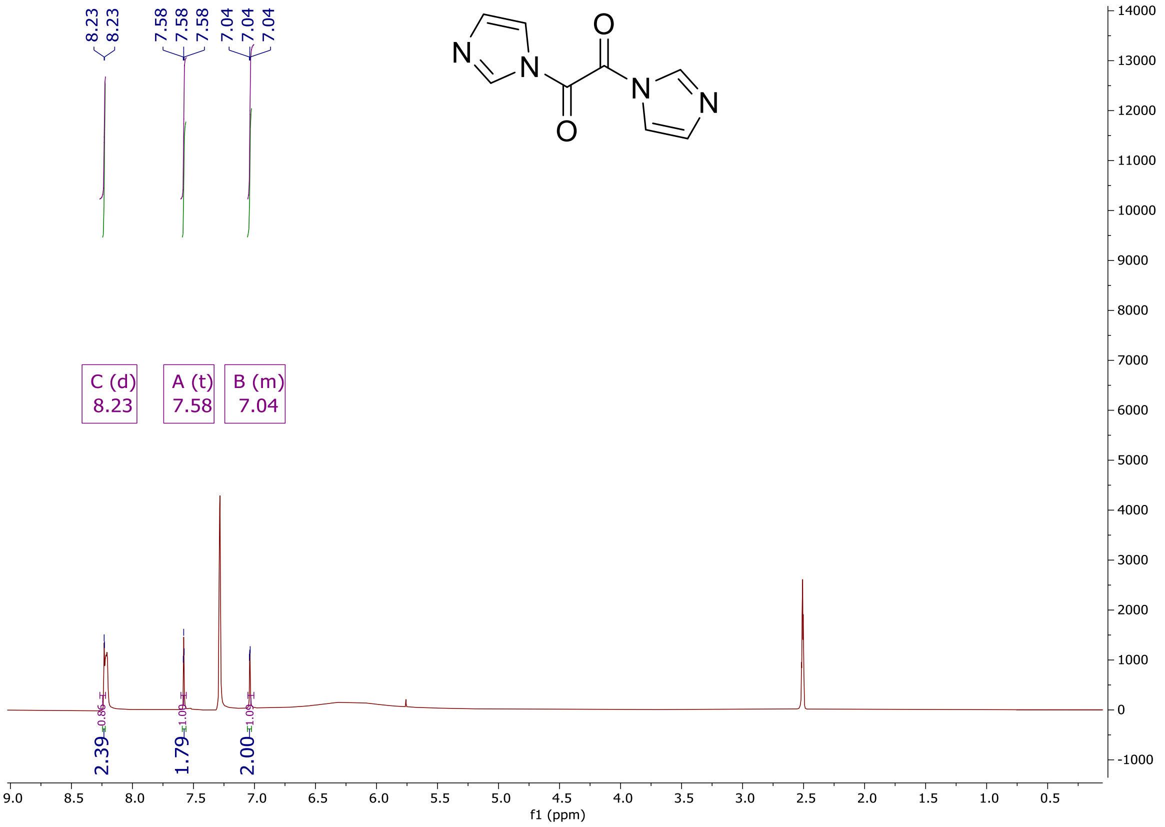
8.23
8.23
7.58
7.58
7.58
7.04
7.04
7.04
C (d) 8.23
A (t) 7.58
B (m) 7.04
2.39
1.79
2.00
f1 (ppm)

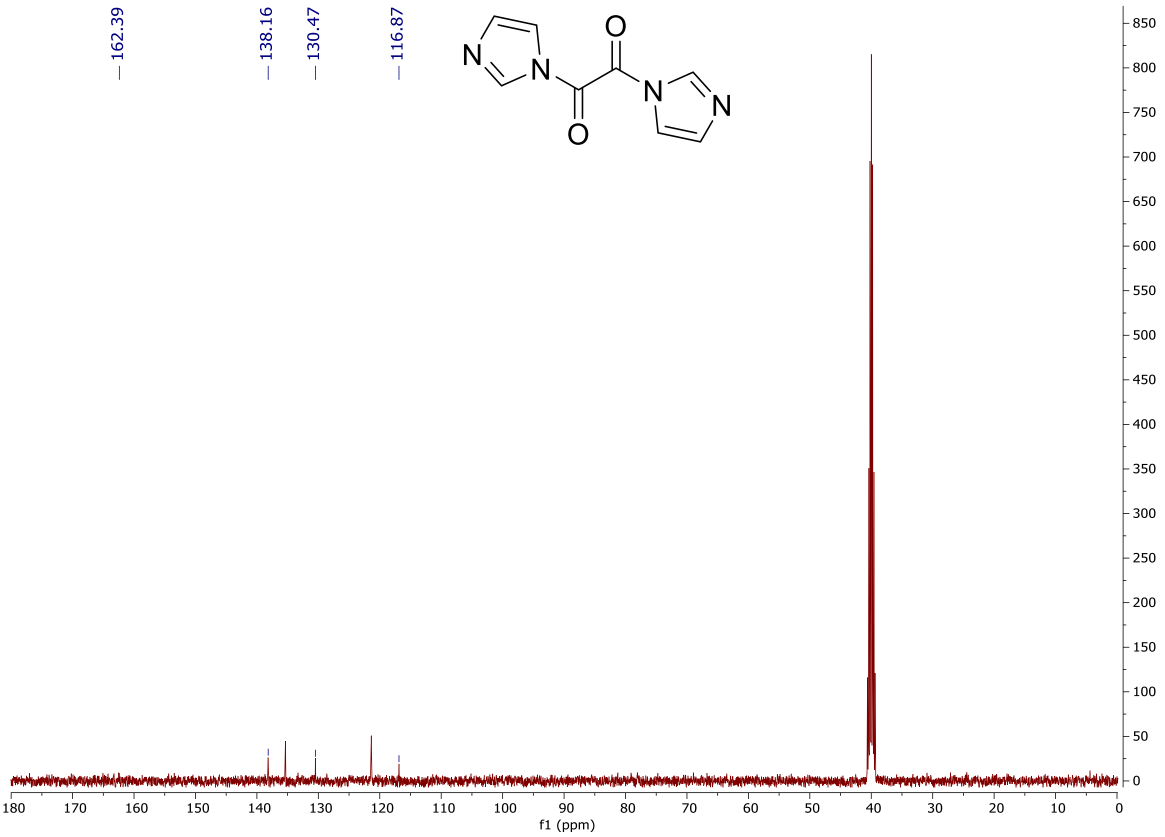
162.39
138.16
130.47
116.87
f1 (ppm)

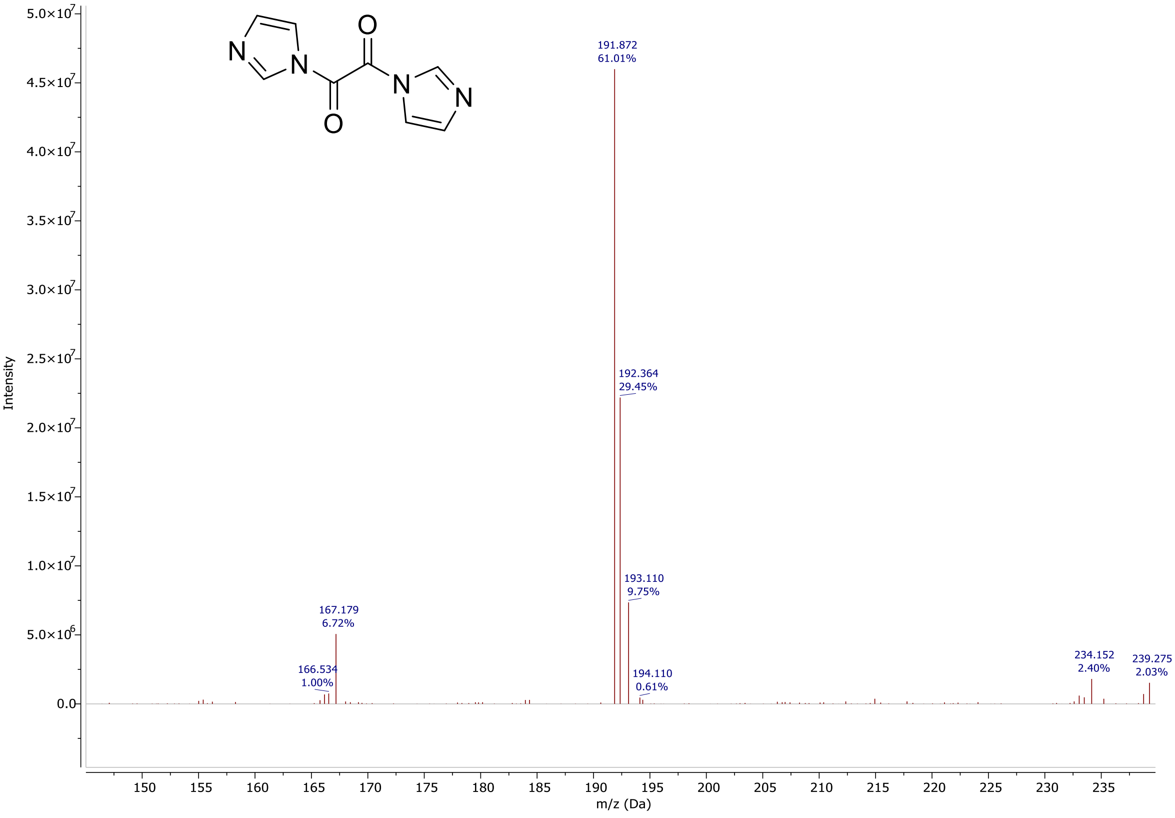
Intensity
5.0×10^7
4.5×10^7
4.0×10^7
3.5×10^7
3.0×10^7
2.5×10^7
2.0×10^7
1.5×10^7
1.0×10^7
5.0×10^6
0.0
191.872
61.01%
192.364
29.45%
193.110
9.75%
194.110
0.61%
167.179
6.72%
166.534
1.00%
234.152
2.40%
239.275
2.03%
150 155 160 165 170 175 180 185 190 195 200 205 210 215 220 225 230 235
m/z (Da)

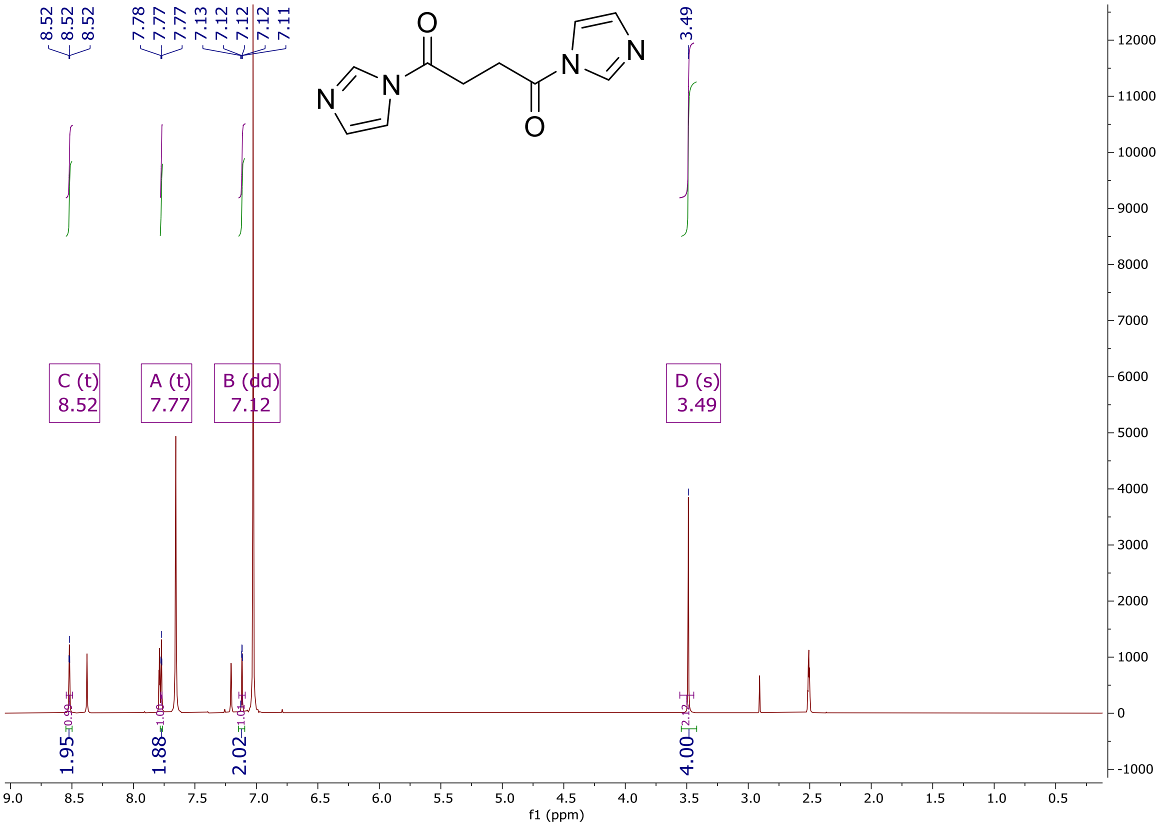
8.52
8.52
8.52
7.78
7.77
7.77
7.13
7.12
7.12
7.12
7.11
3.49
C (t)
8.52
A (t)
7.77
B (dd)
7.12
D (s)
3.49
1.95
1.88
2.02
4.00
9.0 8.5 8.0 7.5 7.0 6.5 6.0 5.5 5.0 4.5 4.0 3.5 3.0 2.5 2.0 1.5 1.0 0.5
f1 (ppm)
12000
11000
10000
9000
8000
7000
6000
5000
4000
3000
2000
1000
0
-1000

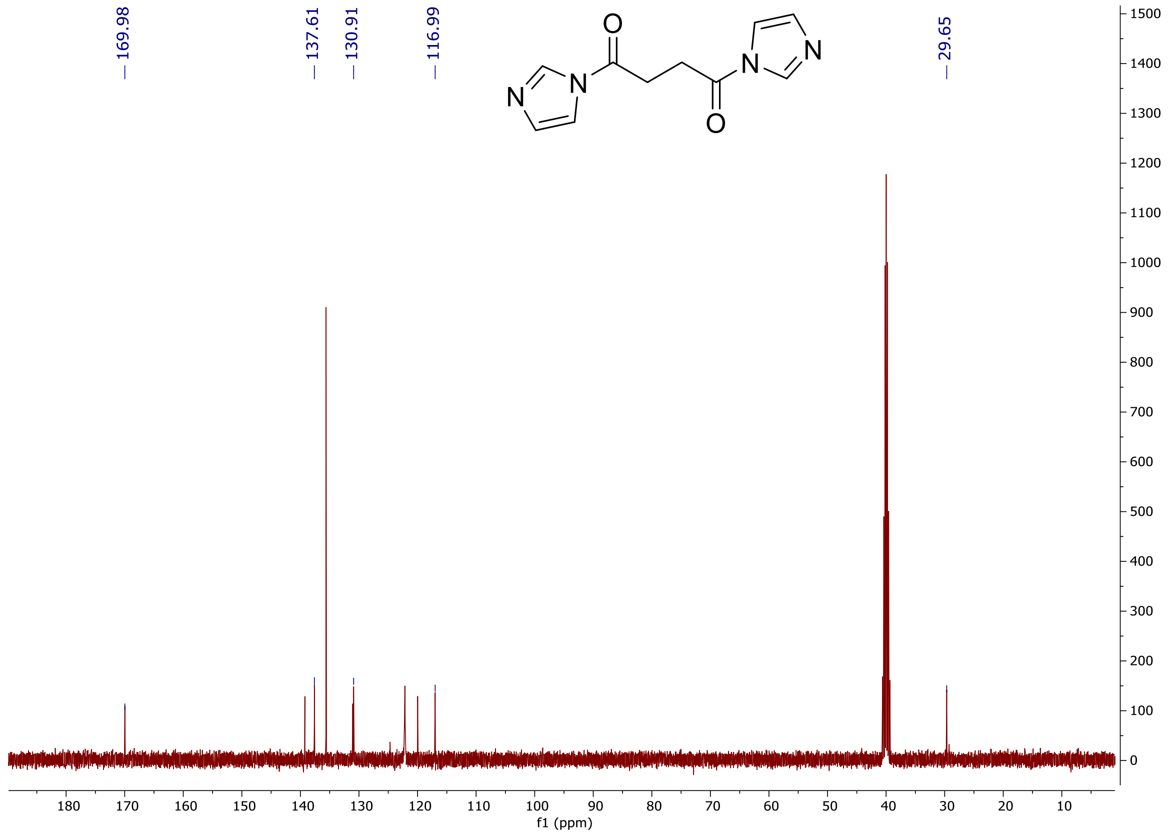

169.98
137.61
130.91
116.99
29.65
O
N
N
N
N
O
f1 (ppm)

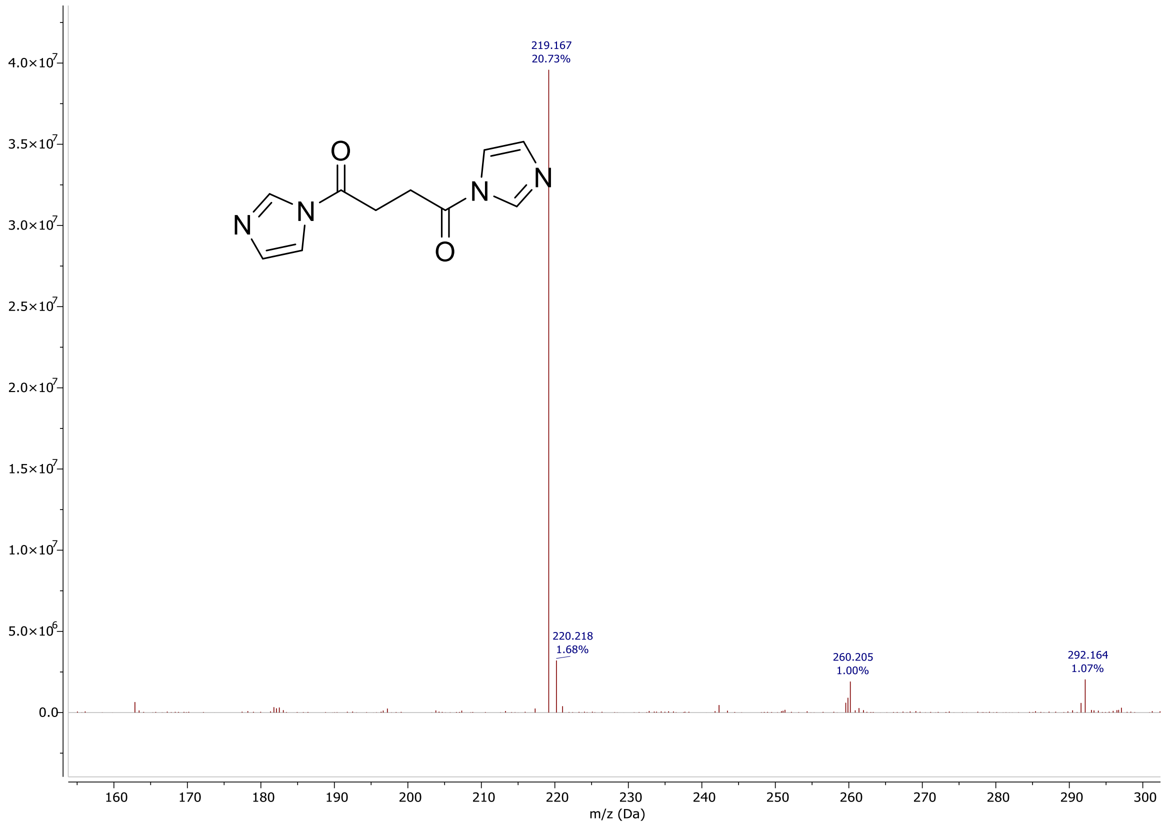

219.167
20.73%
220.218
1.68%
260.205
1.00%
292.164
1.07%
O
N
N
N
N
O
m/z (Da)

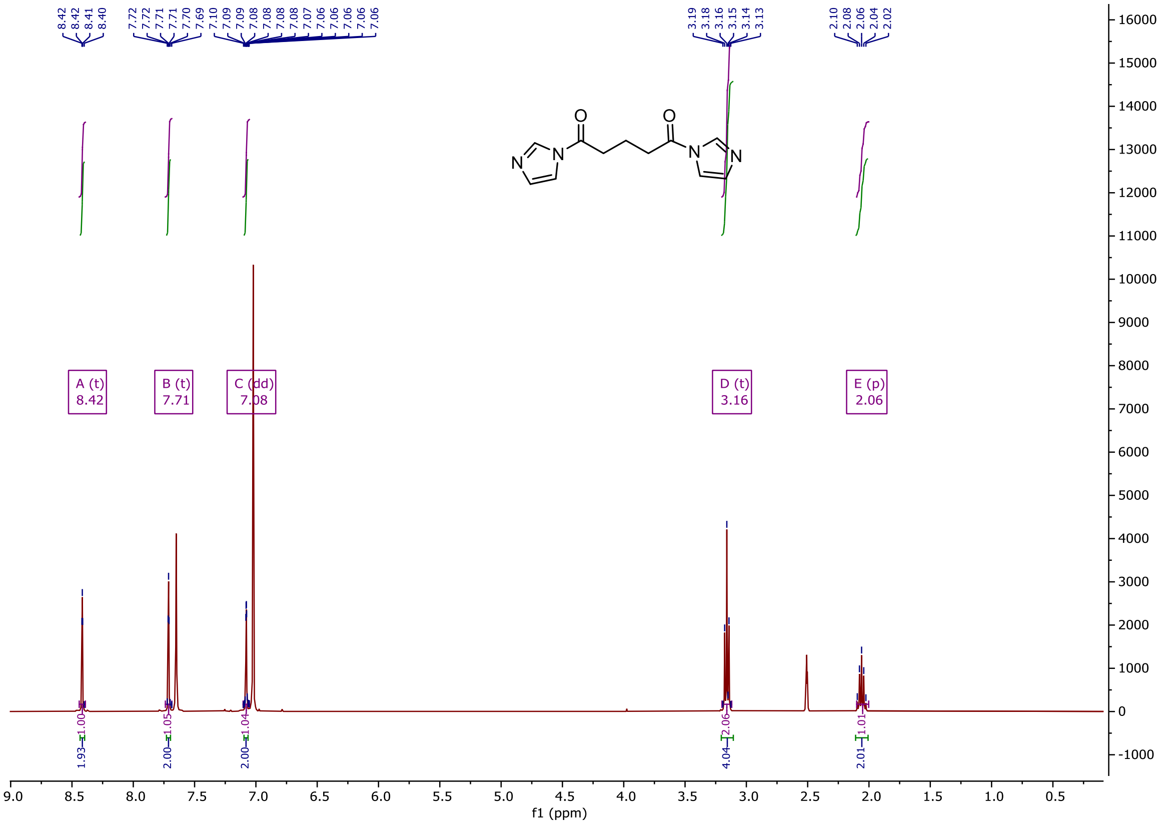
8.42
8.42
8.41
8.40
7.72
7.72
7.71
7.71
7.70
7.69
7.10
7.09
7.09
7.08
7.08
7.08
7.08
7.07
7.06
7.06
7.06
7.06
7.06
3.19
3.18
3.16
3.15
3.14
3.13
2.10
2.08
2.06
2.04
2.02
A (t)
8.42
B (t)
7.71
C (dd)
7.08
D (t)
3.16
E (p)
2.06
1.93
1.00
2.00
1.05
2.00
1.04
4.04
2.06
2.01
1.01
f1 (ppm)

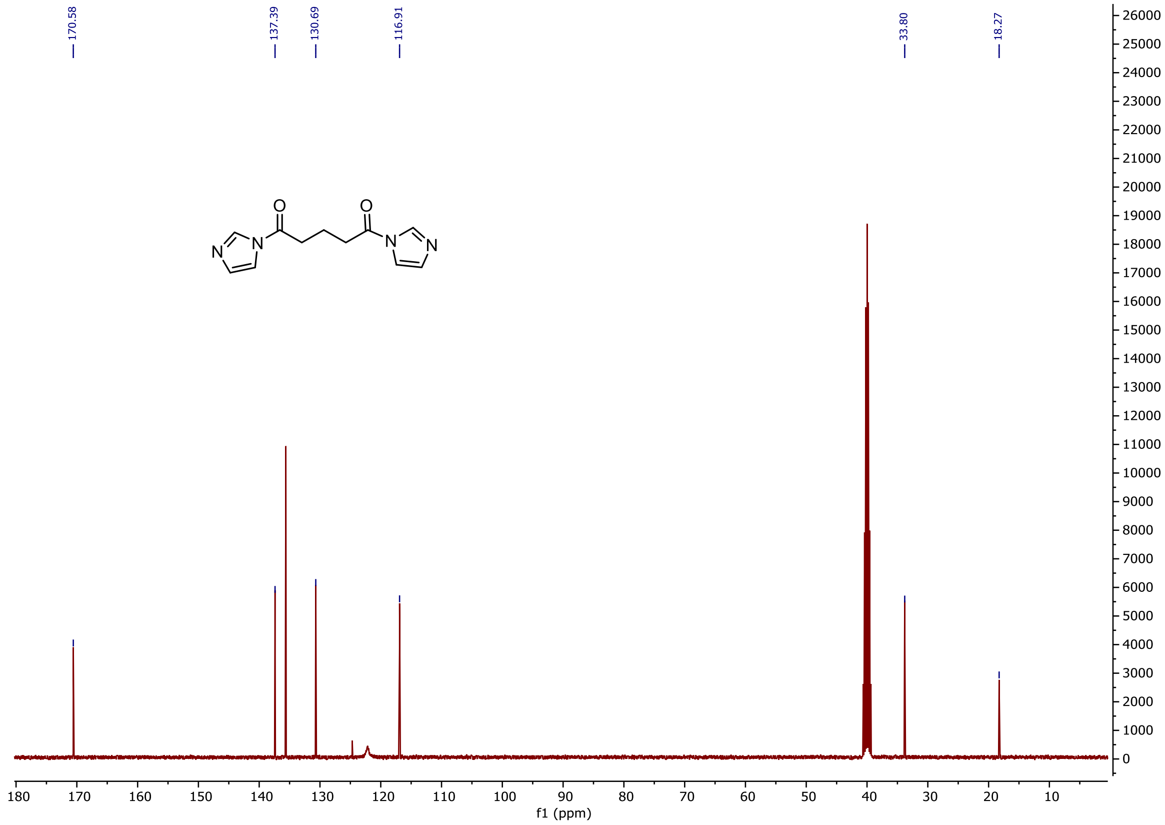
170.58
137.39
130.69
116.91
33.80
18.27
f1 (ppm)

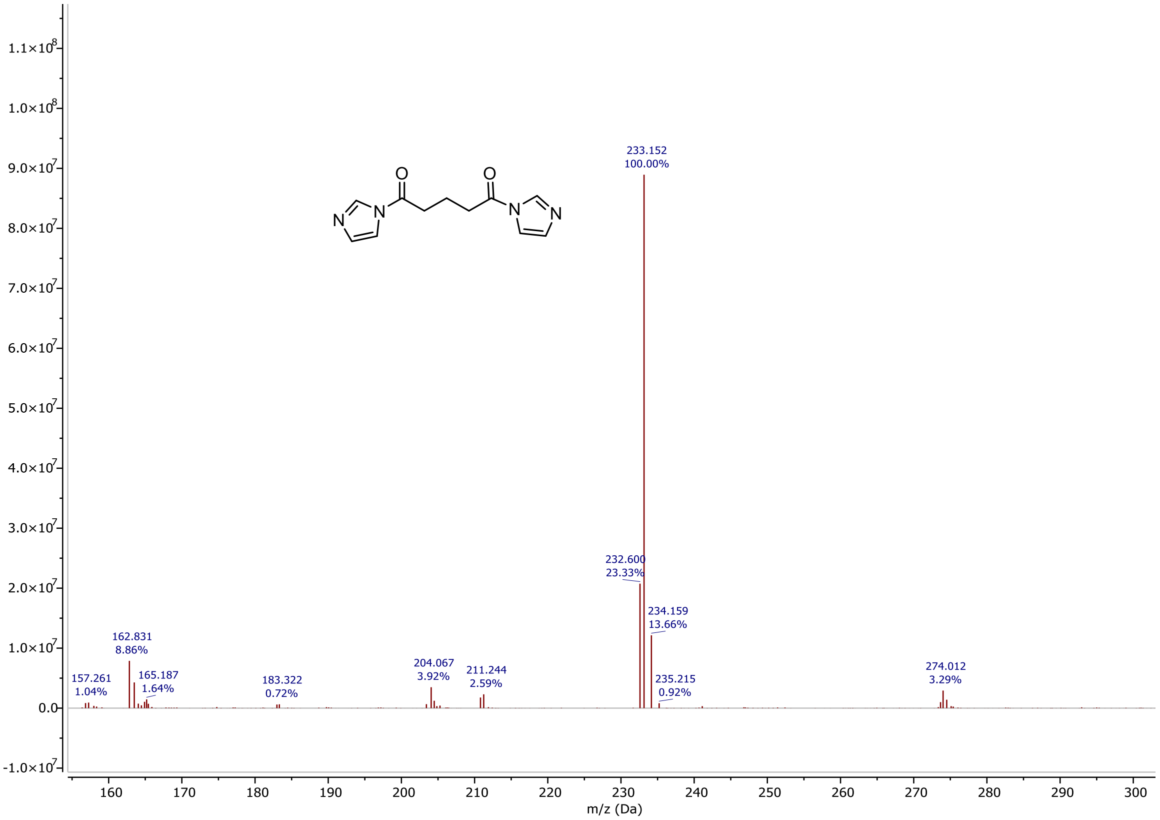
233.152
100.00%
232.600
23.33%
234.159
13.66%
235.215
0.92%
162.831
8.86%
157.261
1.04%
165.187
1.64%
183.322
0.72%
204.067
3.92%
211.244
2.59%
274.012
3.29%
m/z (Da)

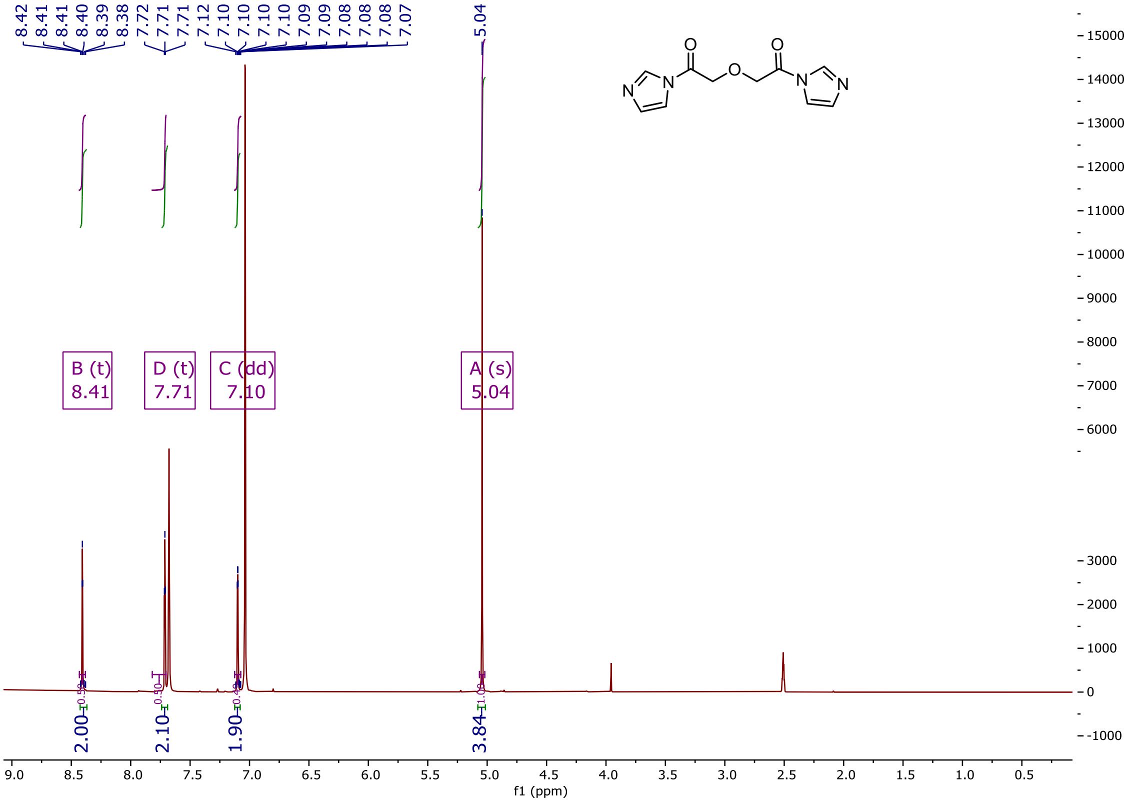
8.42
8.41
8.41
8.40
8.39
8.38
7.72
7.71
7.71
7.12
7.10
7.10
7.10
7.10
7.09
7.09
7.08
7.08
7.07
5.04
B (t)
8.41
D (t)
7.71
C (dd)
7.10
A (s)
5.04
2.00
2.10
1.90
3.84
f1 (ppm)

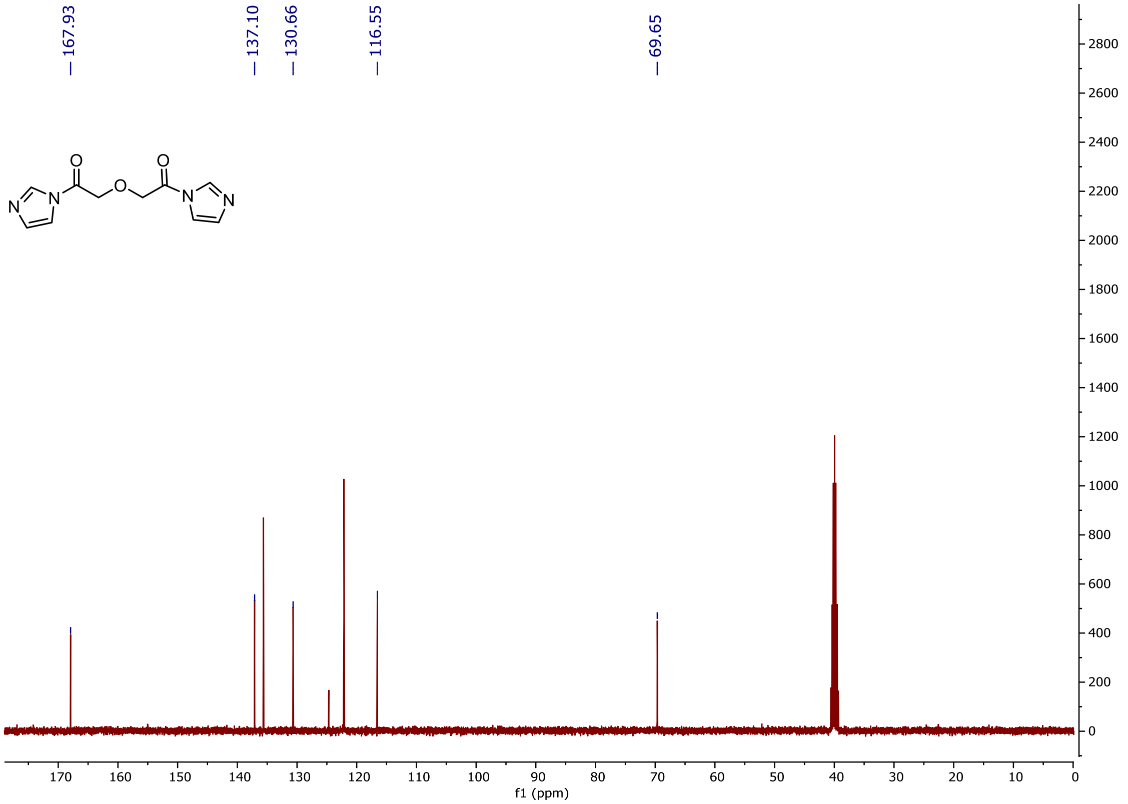

167.93
137.10
130.66
116.55
69.65
f1 (ppm)

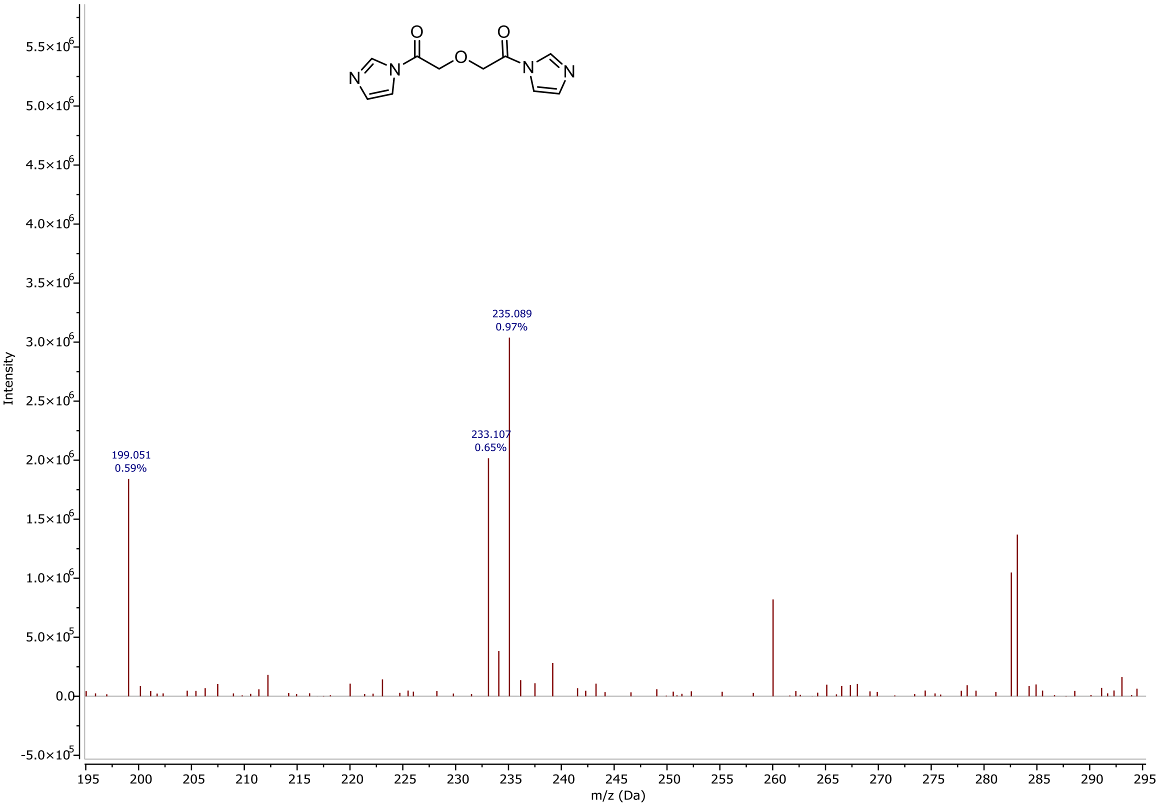

235.089
0.97%
233.107
0.65%
199.051
0.59%
Intensity
m/z (Da)

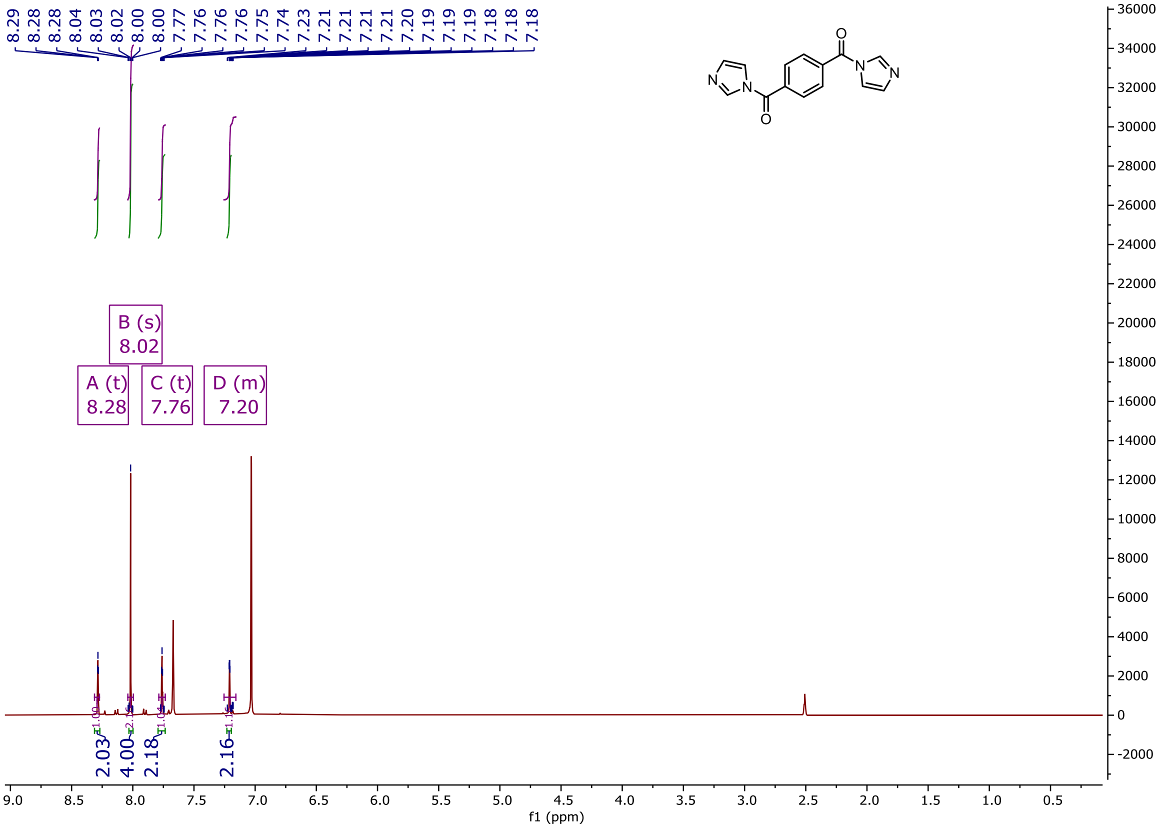

8.29
8.28
8.28
8.04
8.03
8.02
8.00
8.00
7.77
7.76
7.76
7.76
7.75
7.74
7.23
7.21
7.21
7.21
7.21
7.20
7.19
7.19
7.19
7.18
7.18
7.18
N
N
O
O
N
N
B (s)
8.02
A (t)
8.28
C (t)
7.76
D (m)
7.20
2.03
4.00
2.18
2.16
f1 (ppm)

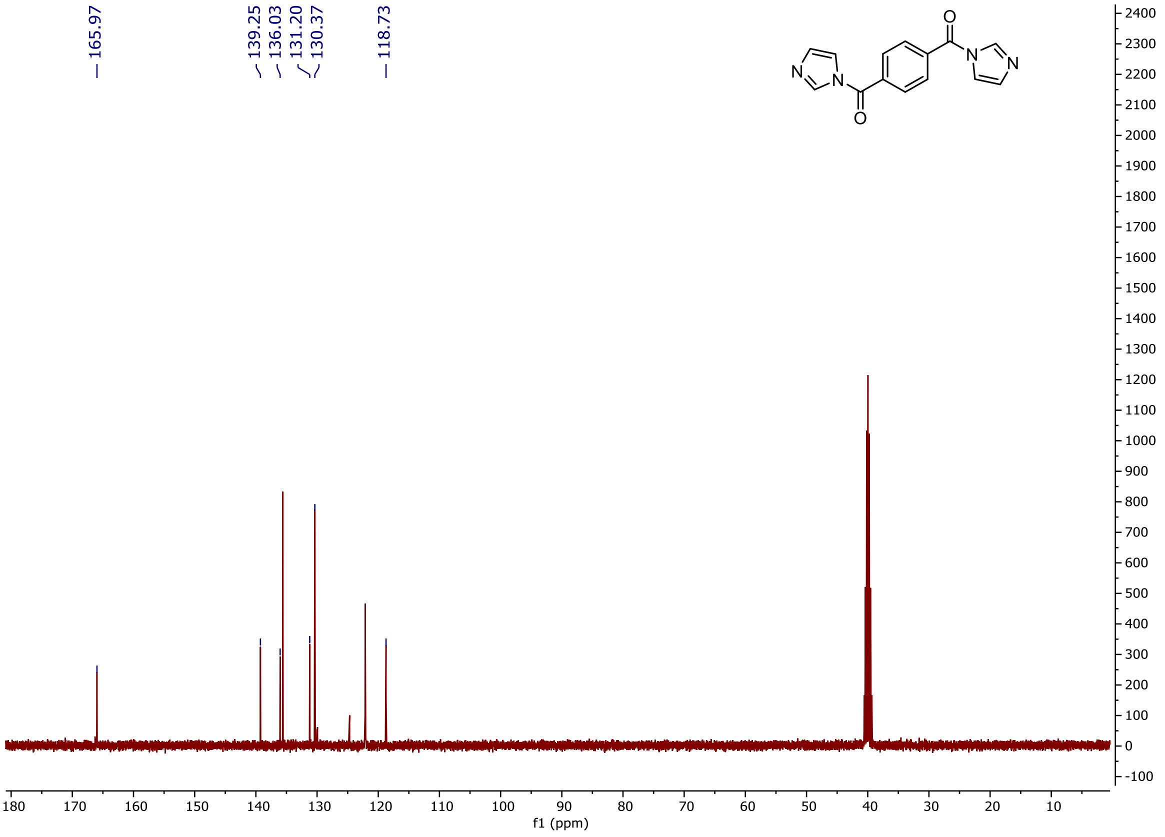

165.97
139.25
136.03
131.20
130.37
118.73
N
N
O
O
N
N
f1 (ppm)

! !

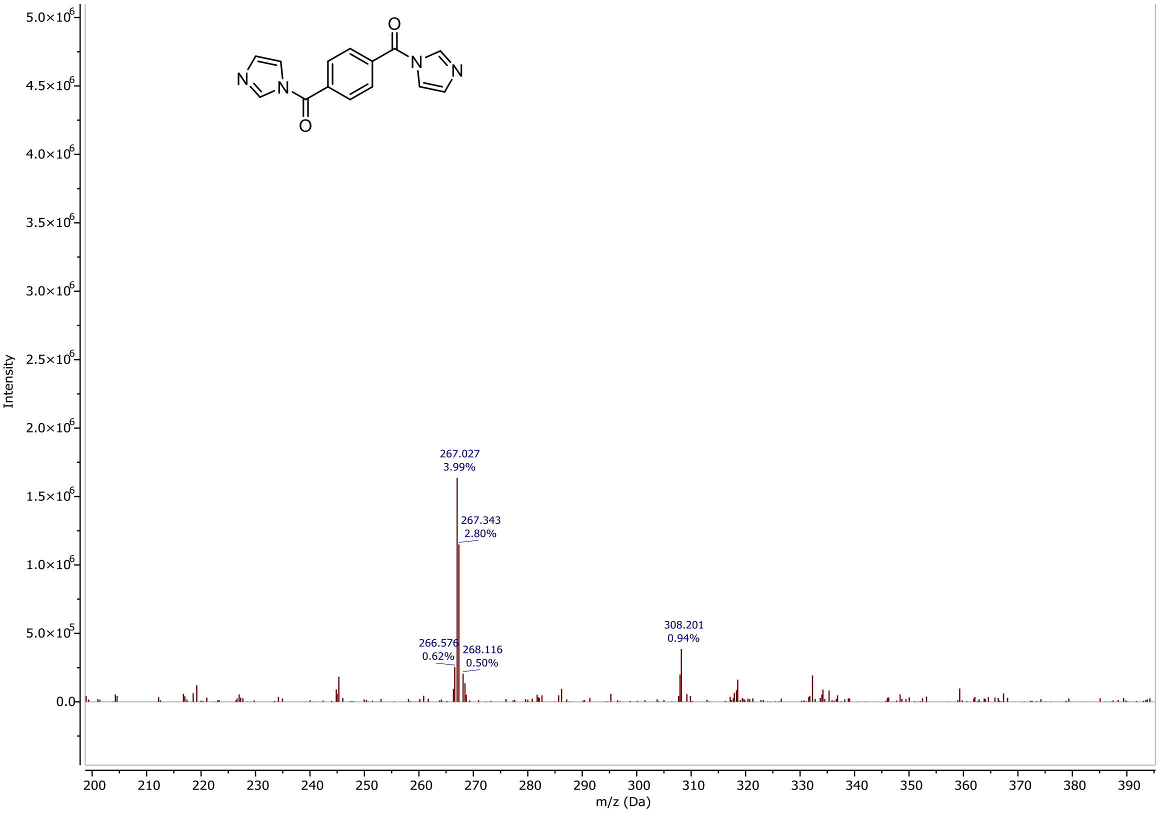

Intensity
m/z (Da)
267.027
3.99%
267.343
2.80%
266.576
0.62%
268.116
0.50%
308.201
0.94%
N
N
O
O
N
N

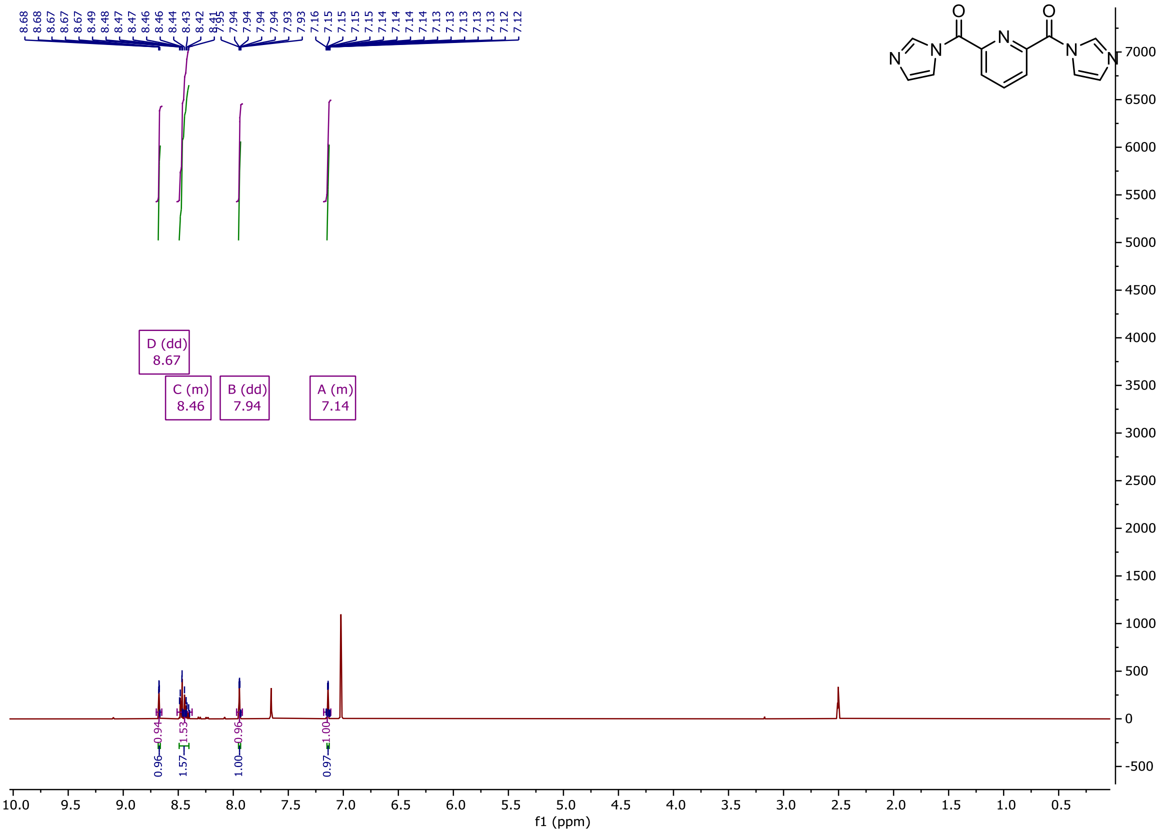

D (dd)
8.67
C (m)
8.46
B (dd)
7.94
A (m)
7.14
0.96
0.94
1.57
1.53
1.00
0.96
0.97
1.00
f1 (ppm)
O
O
N
N
N
N
N

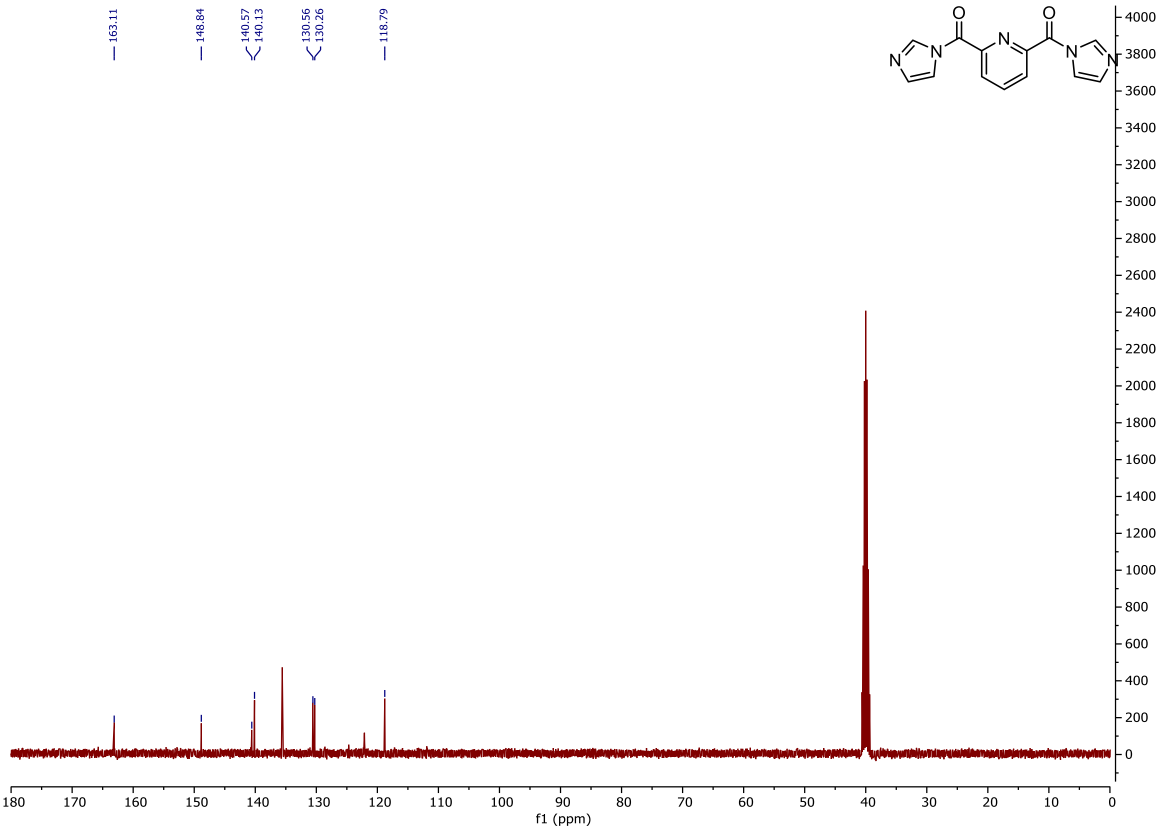
163.11
148.84
140.57
140.13
130.56
130.26
118.79
f1 (ppm)

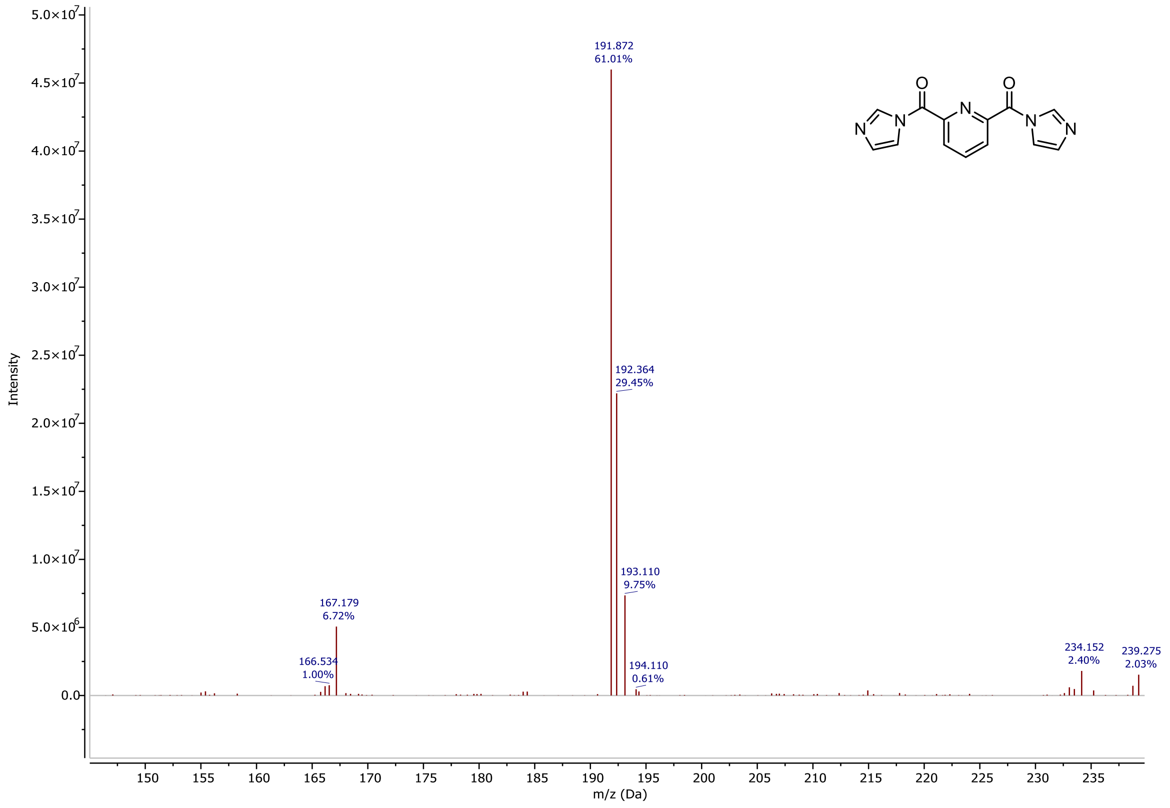
Intensity
m/z (Da)
191.872
61.01%
192.364
29.45%
193.110
9.75%
194.110
0.61%
167.179
6.72%
166.534
1.00%
234.152
2.40%
239.275
2.03%

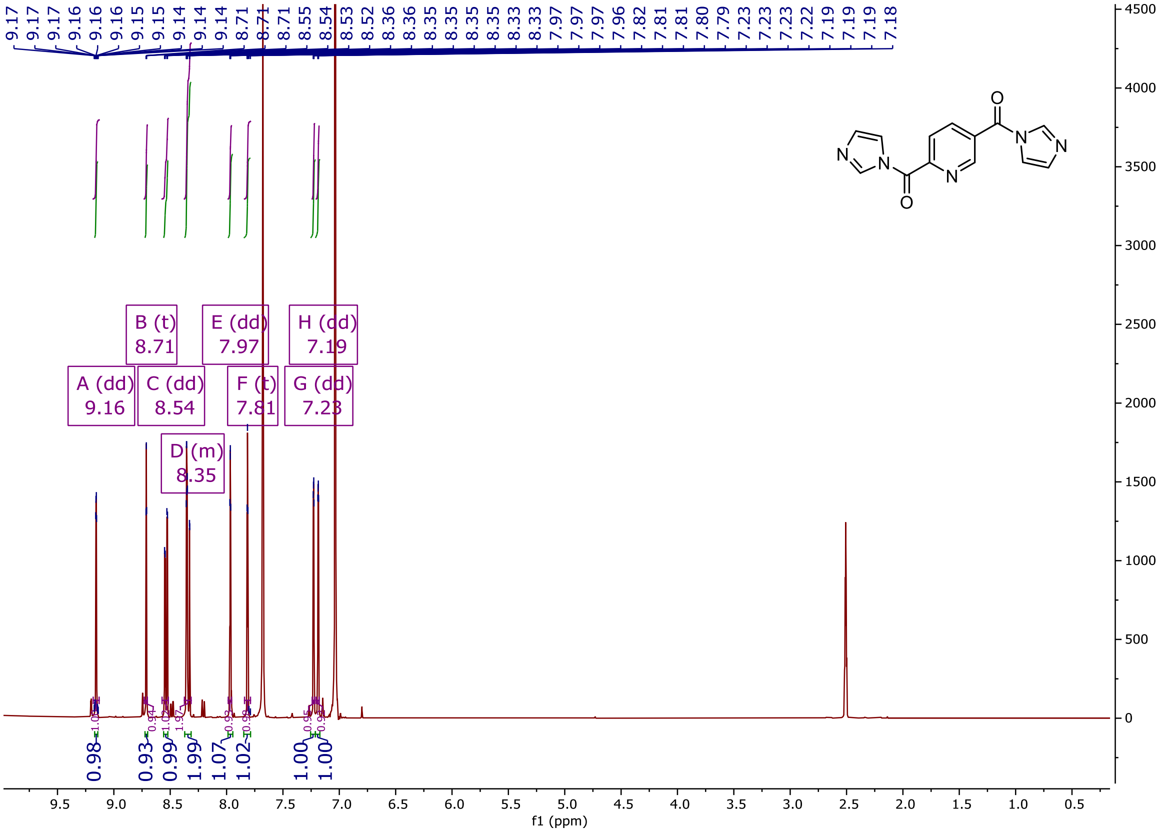

9.17
9.17
9.17
9.16
9.16
9.16
9.15
9.15
9.14
9.14
9.14
8.71
8.71
8.71
8.55
8.54
8.53
8.52
8.36
8.36
8.35
8.35
8.35
8.35
8.33
8.33
7.97
7.97
7.97
7.96
7.82
7.81
7.81
7.80
7.79
7.23
7.23
7.23
7.22
7.19
7.19
7.19
7.18
A (dd)
9.16
B (t)
8.71
C (dd)
8.54
D (m)
8.35
E (dd)
7.97
F (t)
7.81
G (dd)
7.23
H (dd)
7.19
0.98
0.93
0.99
1.99
1.07
1.02
1.00
1.00
f1 (ppm)

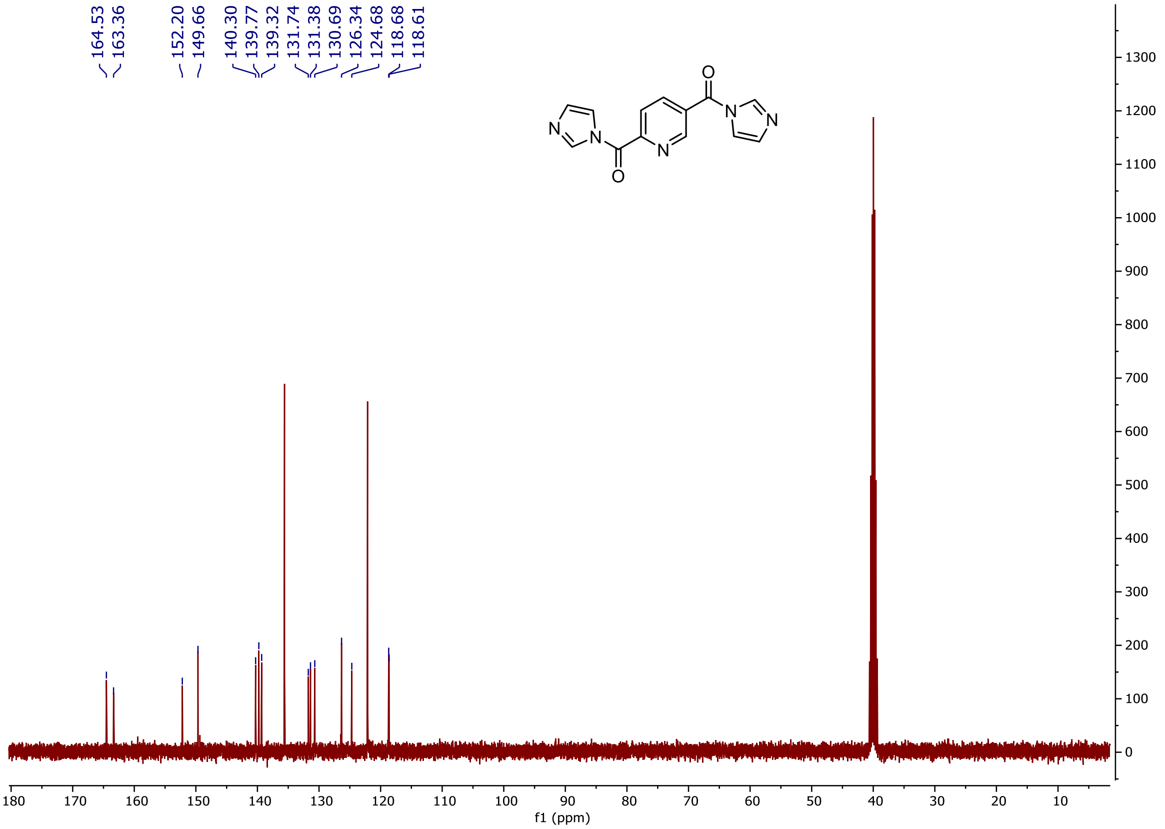

164.53
163.36
152.20
149.66
140.30
139.77
139.32
131.74
131.38
130.69
126.34
124.68
118.68
118.61
f1 (ppm)

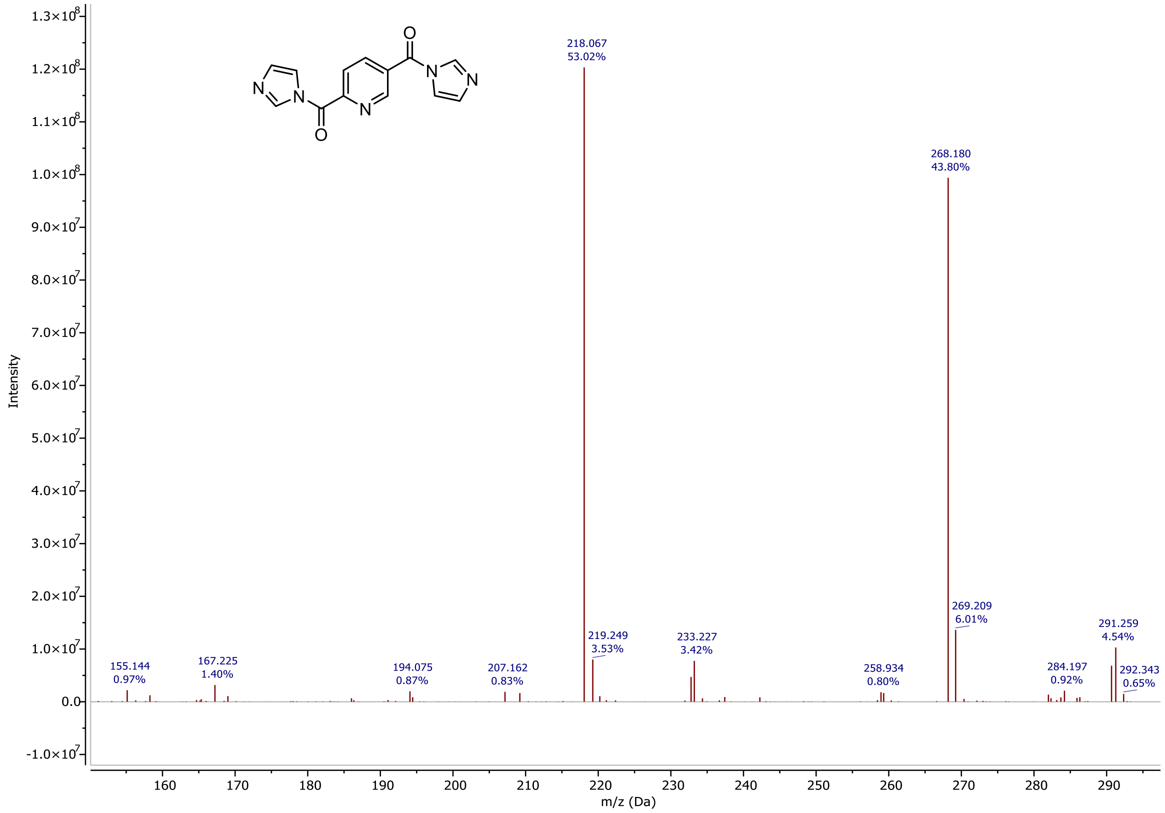

218.067
53.02%
268.180
43.80%
269.209
6.01%
291.259
4.54%
292.343
0.65%
284.197
0.92%
258.934
0.80%
233.227
3.42%
219.249
3.53%
207.162
0.83%
194.075
0.87%
167.225
1.40%
155.144
0.97%
Intensity
m/z (Da)

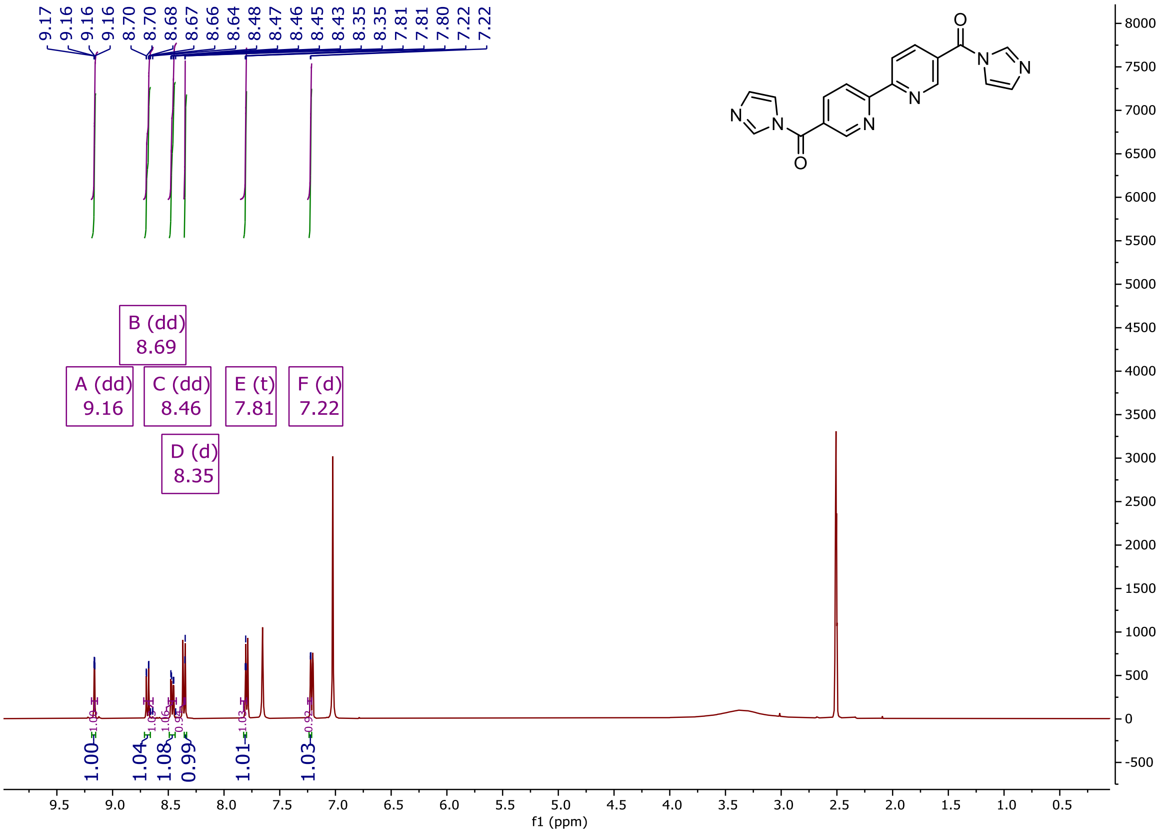

9.17
9.16
9.16
9.16
8.70
8.70
8.68
8.67
8.66
8.64
8.48
8.47
8.46
8.45
8.43
8.35
8.35
7.81
7.81
7.80
7.22
7.22
B (dd)
8.69
A (dd)
9.16
C (dd)
8.46
E (t)
7.81
F (d)
7.22
D (d)
8.35
1.00
1.04
1.08
0.99
1.01
1.03
f1 (ppm)

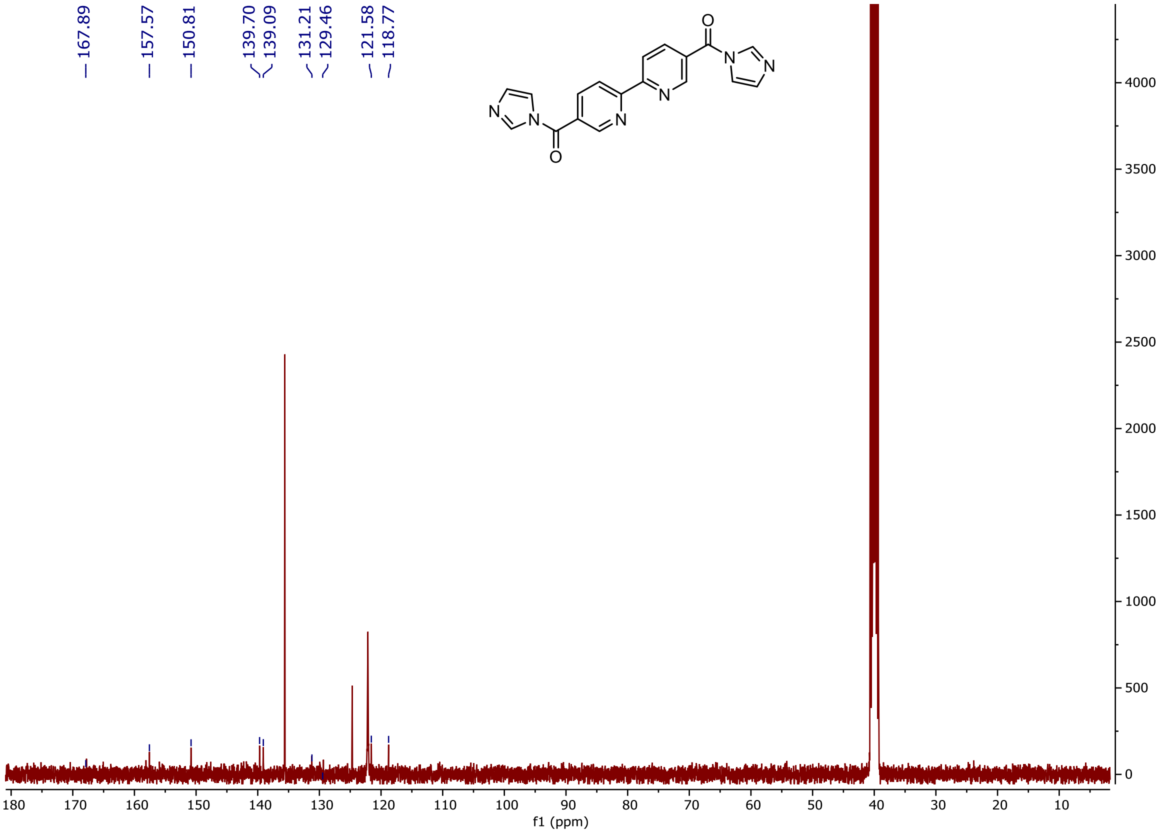
167.89
157.57
150.81
139.70
139.09
131.21
129.46
121.58
118.77
f1 (ppm)

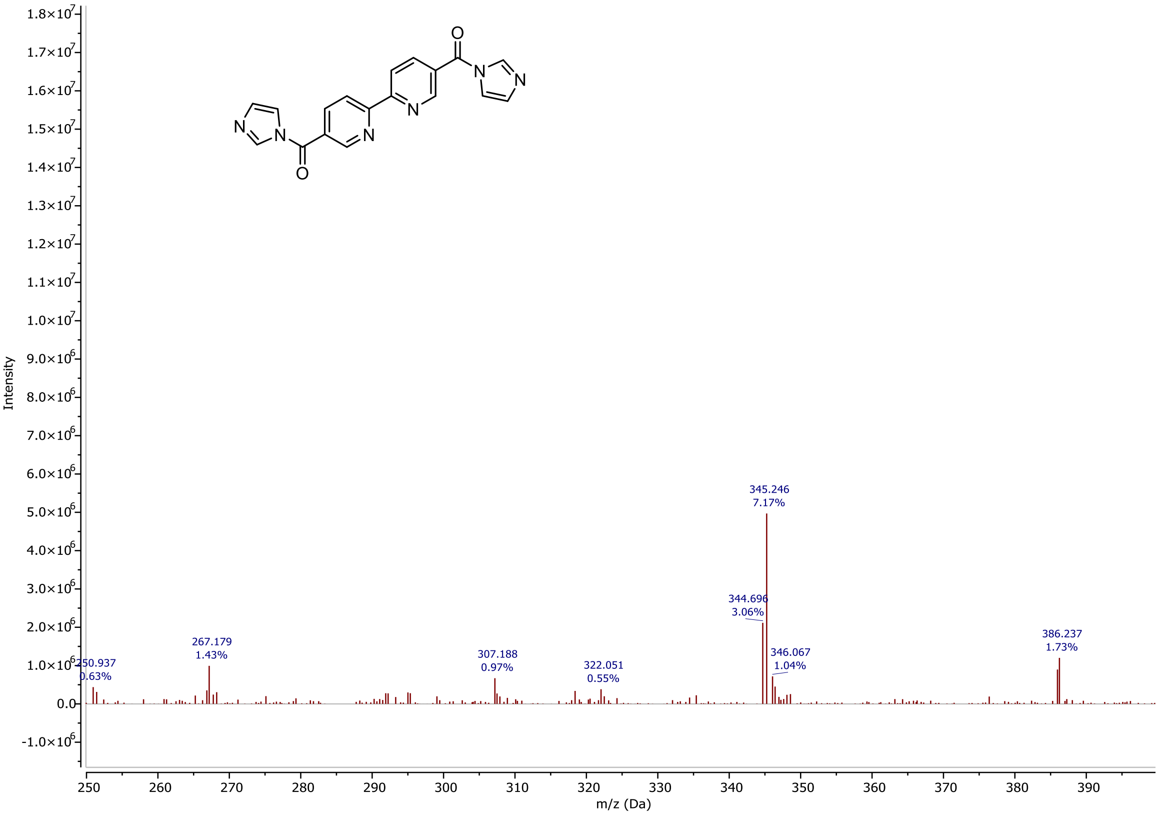
Intensity
m/z (Da)
250.937
0.63%
267.179
1.43%
307.188
0.97%
322.051
0.55%
344.696
3.06%
345.246
7.17%
346.067
1.04%
386.237
1.73%

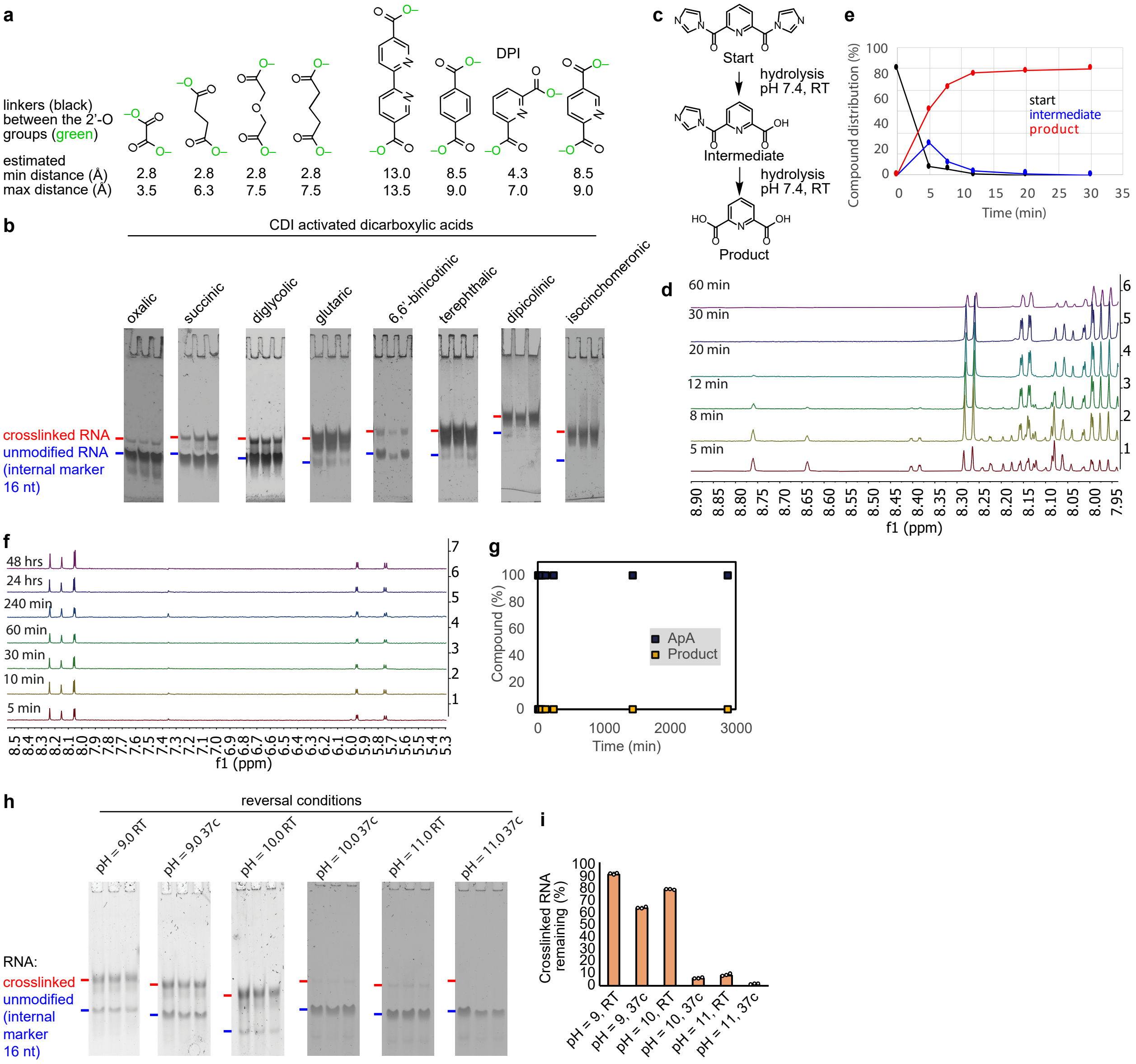

Supplementary Figure 2. Characterization of SHARC chemistry. **a**, Minimal and maximal possible lengths between the crosslinked 2' oxygen groups in RNA, estimated in PyMOL. Hydroxyl groups are represented by the green oxygen atoms. The minimal distances were set at 2.8, approximately the distance between two oxygens between hydrogen-bonded water molecules. **b**, PAGE analysis of crosslinking efficiency with the different activated dicarboxylic acids (100 mM), with 10 µM model RNA 1 in 0.06 M MOPS, pH 7.5; 0.1 M KCl; 2.5 mM MgCl2 at room temperature for 4 hours. The three lanes represent triplicate experiments. **c**, Reaction scheme of hydrolysis reaction of DPI. **d**, Hydrolysis of DPI in phosphate buffer pH 7.4 analysed by NMR spectroscopy over time. **e**, Formation of hydrolyzed DPI products over time. After ~30 min all DPI has been hydrolyzed. **f**, Hydrolysis of ApA in 100 mM borate buffer pH 10.0 analysed by NMR spectroscopy over time. **g**, Formation of hydrolyzed ApA products over time, based on quantification of panel f. Even after 48 hours, no hydrolysis products are observed. **h**, PAGE analysis of crosslink reversal efficiency at different alkaline and temperature conditions (37 C or room temperature RT). The three lanes represent triplicate experiments. **i**, An increase in crosslink reversal (= decrease in crosslinked RNA) is observed at increased pH and temperature, based on quantification of gel pictures in panel h. Near complete reversal was achieved at pH 10-11 without obvious damage. Data are mean ± s.d.; n = 3, technical replicates. Source data are provided as a Source Data file.

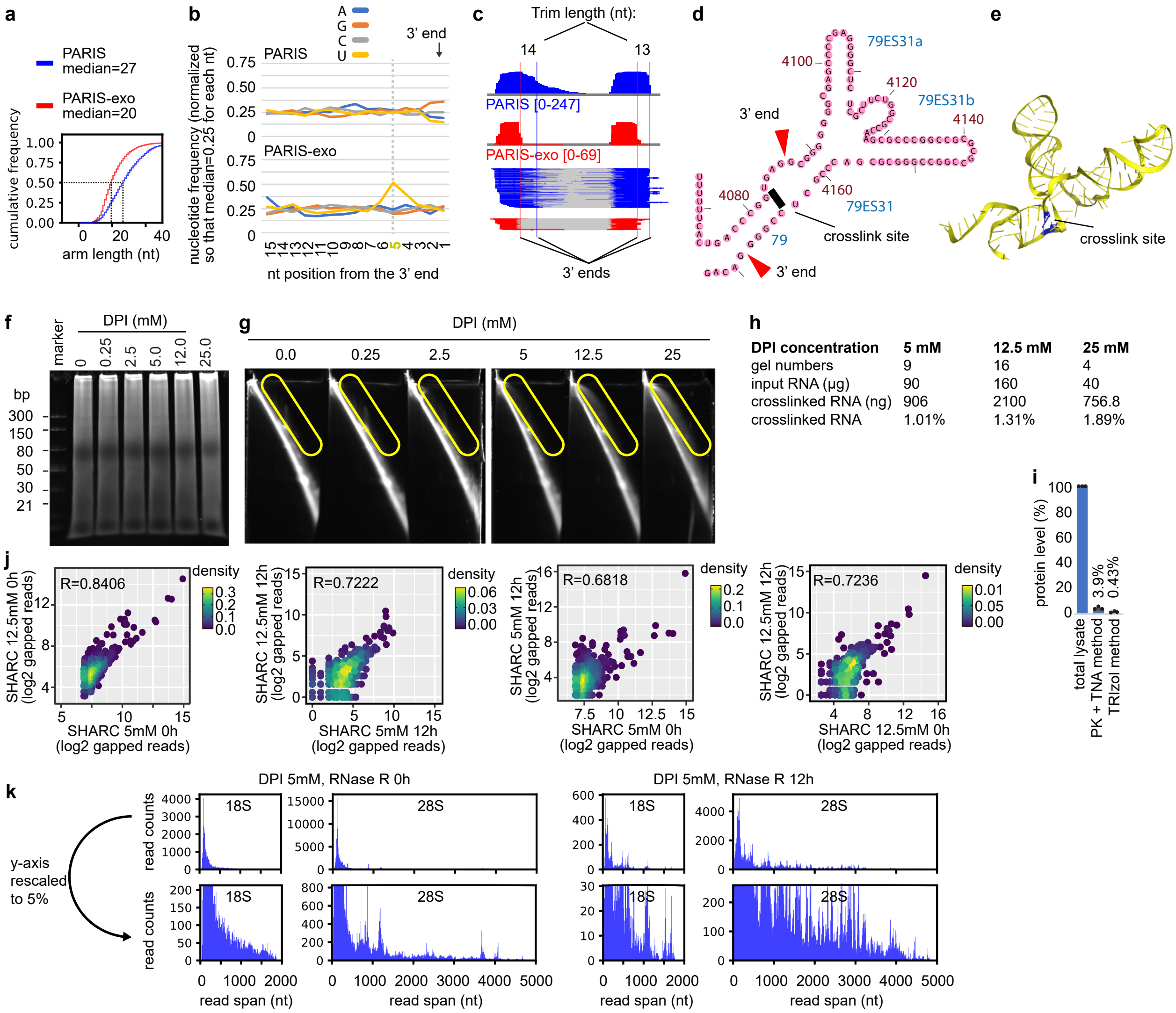

DPI concentration	5 mM	12.5 mM	25 mM
gel numbers	9	16	4
input RNA (µg)	90	160	40
crosslinked RNA (ng)	906	2100	756.8
crosslinked RNA	1.01%	1.31%	1.89%

Supplementary Figure 3. Establishing the exo method for PARIS (a-e) and SHARC (f-j). a, RNase R reduces arm length in PARIS-exo. **b,** RNase R trimming leads to enrichment of U at the 5th nucleotide from the 3' end. **c-e,** An example of precise crosslink site identification using PARIS-exo. **c**, Blue and red vertical lines indicate the 3' ends (median) from PARIS and PARIS-exo reads. **d,** The PARIS-exo derived 3' ends and crosslinking sites are mapped to the H79 and ES31 of human 28S secondary structure. **e,** cryo-EM structure of H79 and ES31 (PDB: 4V6X). **f**, HEK293 cells are crosslinked with DPI at different concentrations. Total RNA crosslinked cells are fragmented by RNase III and separated on a 8% denatured urea-TBE gel. **g**, RNA fragments from the first dimension (panel f) were electrophoresed again on a second dimension of 16% urea-TBE gel. The smear above the diagonal represents crosslinked RNA. **h**, Quantification of the recovery of crosslinked RNA fragments from the DD2D gel system (replicates n = 9, 16 and 4 for the 3 conditions, respectively). The increase in yield is not linear in response to higher crosslinker concentration, because most accessible crosslinking sites have reacted at lower concentrations, yielding an increase in concentration ineffective. **i,** To measure protein content in crosslinked and purified RNA samples, we first crosslinked cells with 5mM DPI. We prepared, in triplicates, (1) total cell lysate in RIPA buffer, (2) RNA extracted using the PK and TNA method, and (3) RNA extracted using standard TRIzol method. All samples were measured for protein concentration using the BCA method, and values normalized against the total lysate. Relative protein concentrations for the PK+TNA method: 4.66%, 3.58%, 3.41%. Relative protein concentrations for the TRIzol method: 0.28%, 0.66%, 0.34%. Data are mean ± s.d.; n = 3, biological replicates. **j**, Scatter plot of gapped reads in each duplex group (DG) among experiments with different conditions. n=5000 genes. **k**, The span of SHARC (5mM DPI, no RNase R trimming) and SHARC-exo (5mM DPI and 12 hour RNase R) gapped reads mapped to the ribosomal RNAs 18S (1869nt) and 28S (5070nt). The lower panels are the same data as upper, with the y-axis rescaled to 5% to show the longer-distance reads. Source data are provided as a Source Data file.

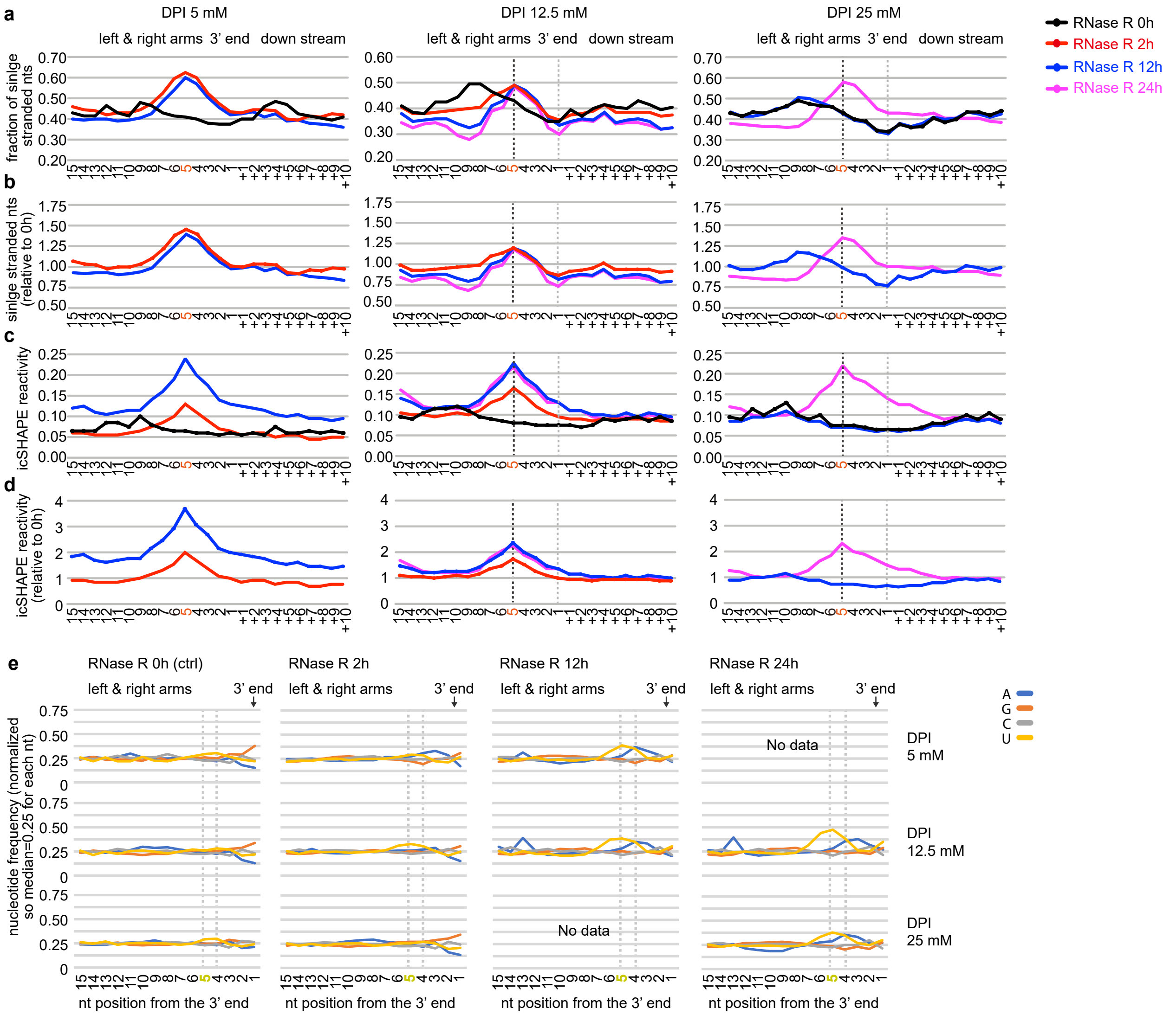

Supplementary Figure 4. SHARC crosslinking and RNase R trimming reveal crosslink sites with high resolution. The 28S rRNA was analyzed for all SHARC experiments. **a,** Fraction of single-stranded nucleotides around the 3' end of the left and right arms. Single stranded nucleotides were defined based on the human ribosome cryo-EM structure (PDB: 4V6X). RNase R trimming leads to a dramatic enrichment of single-stranded nucleotides (ss-nts) around the 5th nucleotide, marked by the black vertical dashed line. **b,** Quantification of differences between RNase R trimmed samples vs. non-trimmed PARIS or SHARC data shown in panel **a**, at the 5th nucleotide position upstream of the 3' end. **c,** Average icSHAPE reactivity around the 3' end of the left and right arms, showing better enrichment of accessible nucleotides at the 5th position than panel **a**. **d,** Quantification of differences between RNase R trimmed samples vs. non-trimmed SHARC data shown in panel **c**, at the 5th nucleotide position upstream of the 3' end. In panels a and c, the higher signal for the non-trimmed SHARC data between 7 and 15 reflects the diffused higher probability single stranded regions. This higher signal collapsed around the 5th nucleotide upon RNase R trimming. **a-d**, n=2 biological replicates. **e**, Frequencies of the 4 nucleotides around the 3' end of the left and right arms. Stronger RNase R trimming revealed a slight enrichment of A and U near the 5th nucleotide, likely reflecting the weaker secondary structure constraints near the SHARC crosslinking sites. Source data are provided as a Source Data file.

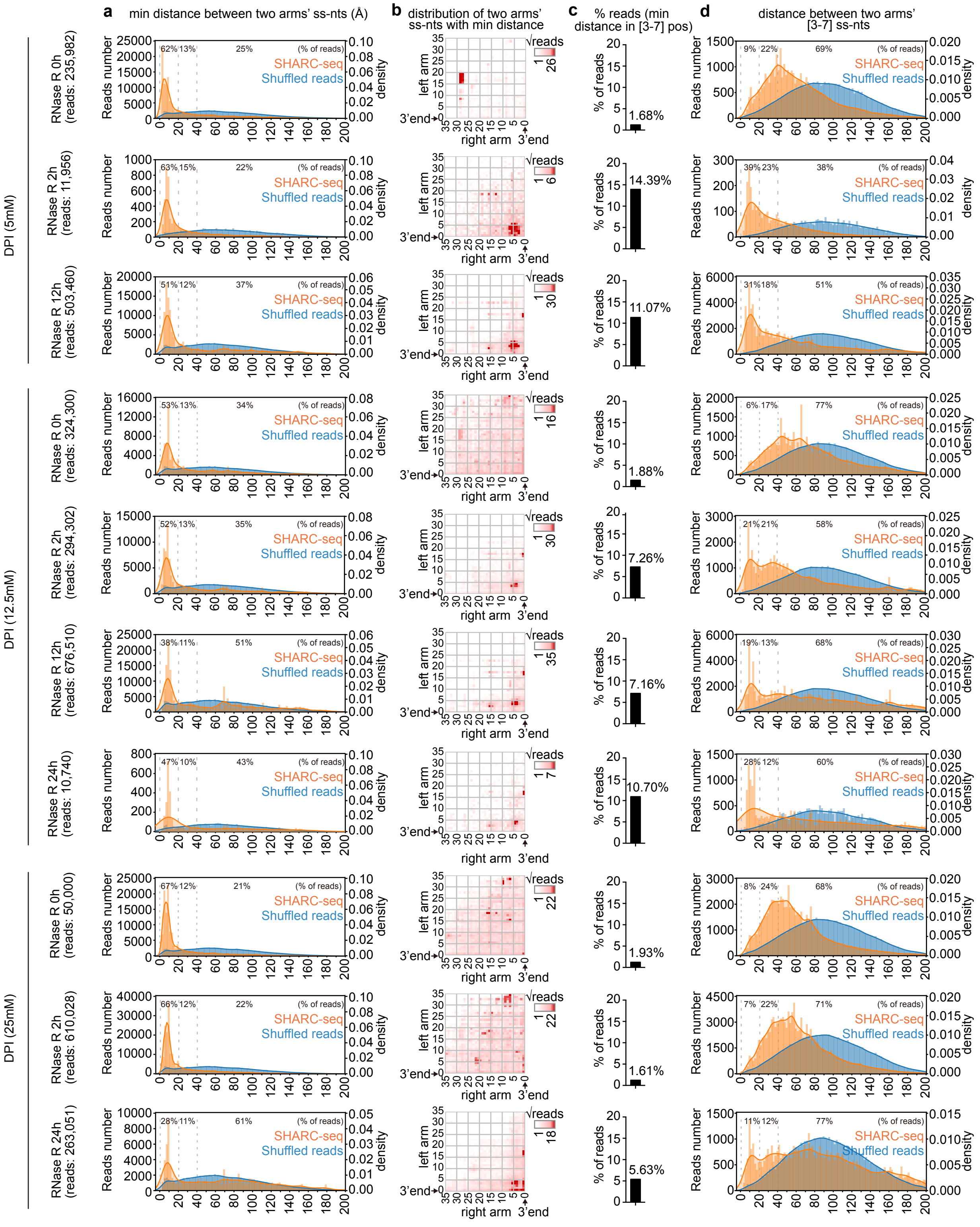

Supplementary Figure 5. Characterization of SHARC crosslinking and RNase R trimming conditions. a, The distribution of minimal distances between two arms' ss-nts (single-stranded nucleotides). **b**, Heatmap showing the positions of two arms' ss-nts at min distance; **c**, Percentage of reads, with minimal distance located in [3,7] x [3,7] positions. **d**, The distribution of distances between the two arms' 3rd to 7th ss-nts. In panels a and d, kernel distributions are represented by lines and labeled on the right; percentages of reads with distances in 0-20, 20-40 and >40 Angstroms. Source data are provided as a Source Data file.

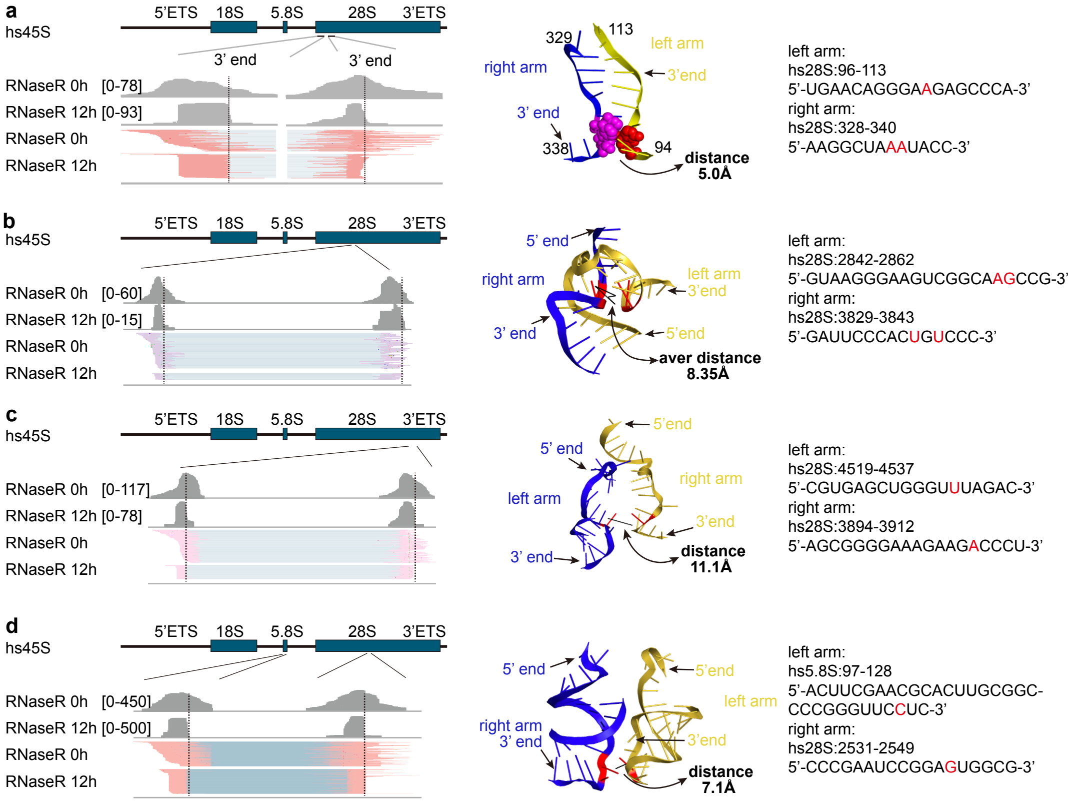

Supplementary Figure 6. Example secondary and tertiary proximal nucleotides crosslinked by SHARC-exo in the ribosome. SHARC sequencing data for the ribosome with or without RNase R treatment are compared (0 vs. 12 hour RNase R) on the left panels. Vertical dash lines represent the median 3' ends for the RNase R trimmed reads. In the middle, positions of 3' ends, crosslinking sites and distances are labeled on the cryo-EM model of the ribosome (PDB:4V6X). Sequences of the region are shown on the right. Panels **a-b** are examples of spatial proximity constrained by secondary structures. Panels **c-d** are examples of tertiary contacts either within (**c**) or between (**d**) RNA molecules. Source data are provided as a Source Data file.

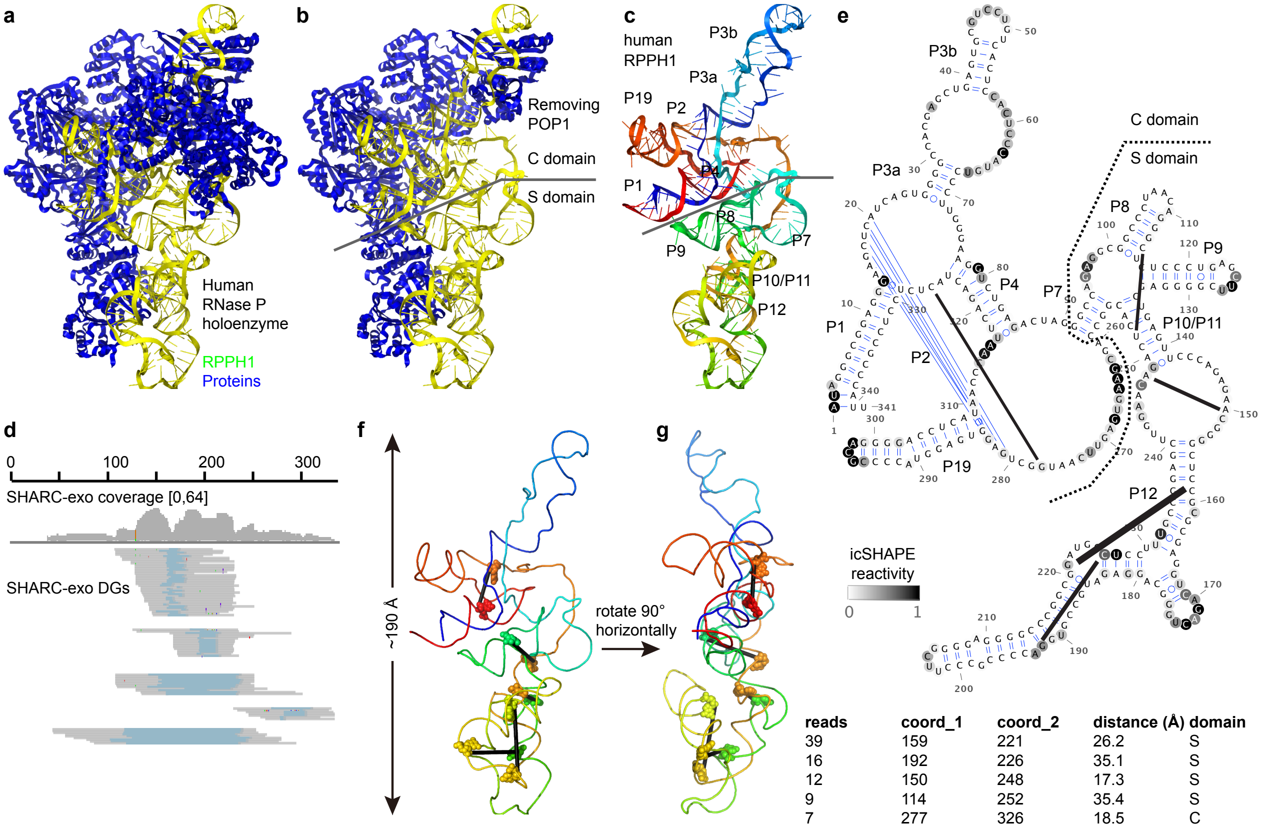

reads	coord_1	coord_2	distance (Å)	domain
39	159	221	26.2	S
16	192	226	35.1	S
12	150	248	17.3	S
9	114	252	35.4	S
7	277	326	18.5	C

Supplementary Figure 7. SHARC-exo captures spatial distances in the RPPH1 RNA in human RNase P. a, Cryo-EM structure of the RNase P holoenzyme, which contains 1 RNA and 10 protein partners (PDB: 6AHR). **b**, The holoenzyme contains an RNA core, the C domain, stabilized by extensive tertiary interactions, and the S domain, which is largely exposed and potentially dynamic (The removed protein POP1 is chain B in 6AHR). **c**, Helices in RPPH1: P1-P19, color-coded blue to red from the 5' end to the 3' end. **d**, SHARC-exo data showing all DGs with >5 reads each. **e**, icSHAPE and SHARC-exo (black lines) data overlapped onto the secondary structure of the RPPH1 RNA. icSHAPE data were extracted from our recent study (Lu et al. 2016 Cell, PMID: 27180905). Thickness of the black lines are scaled to the square root of the read numbers shown at the bottom. Coord_1 and coord_2 are the two crosslinked nucleotides in each DG. **f-g**, Crosslinking sites mapped to the 3D structure of RPPH1, in two views rotated horizontally. Crosslinked nucleotides are shown in spheres. A straight black line was drawn between each pair of nucleotides at the 2'O positions. Source data are provided as a Source Data file.

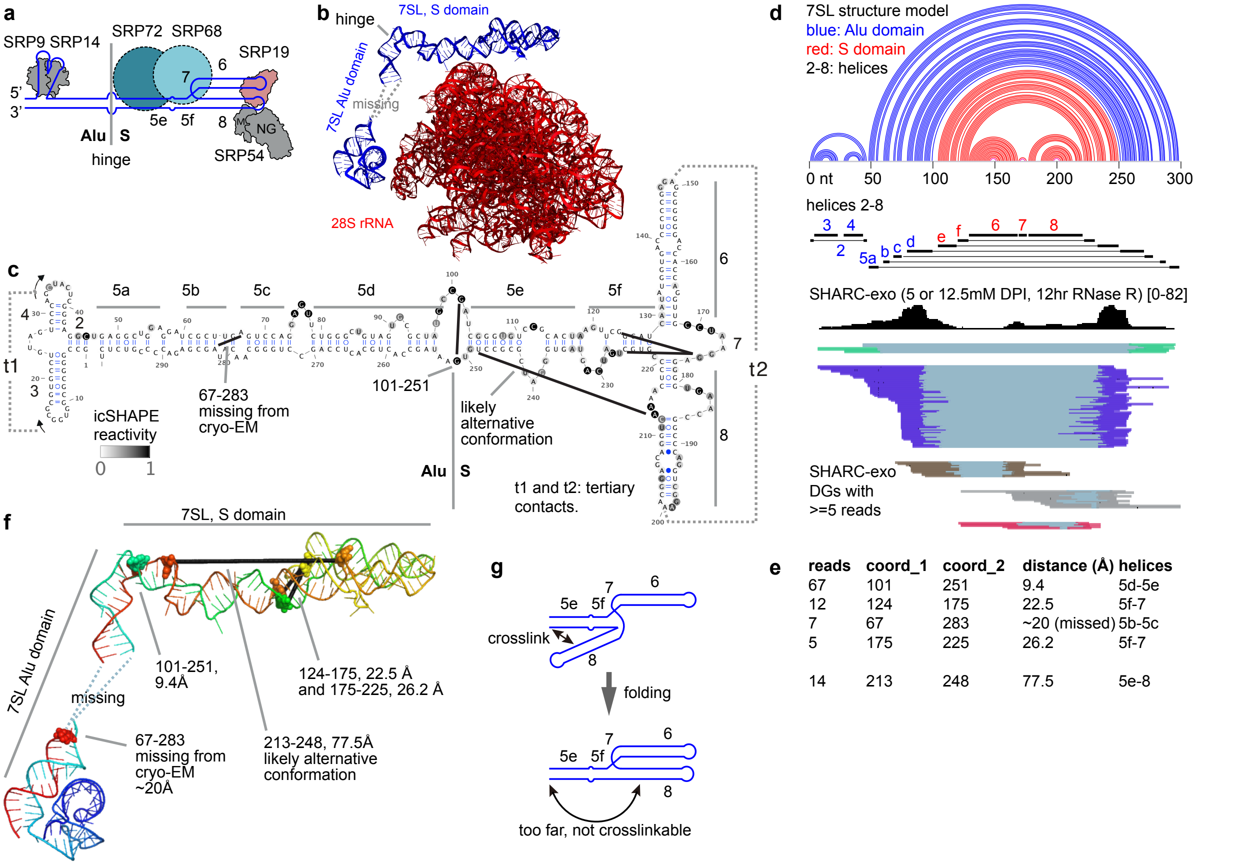

Supplementary Figure 8. SHARC-exo measures spatial distances in the 7SL RNA. **a**. Model of the SRP complex, which consists of the 7SL RNA and 6 proteins. These components can be organized into 2 major domains, Alu and S, separated by the hinge/elbow. The Alu domain contains helices 2, 3, 4, 5a, 5b, 5c, 5d, and proteins SRP9/14. The S domain contains helices 5e, 5f, 6, 7, 8, and proteins SRP19/54 and SRP68/72, where the SRP54 recognizes nascent peptides from ribosome. Redrawn from Grotwinkel et al. 2014 (PMID: 24700861). **b**. Cryo-EM structure of the 7SL RNA on the 28S rRNA (PDB: 6FRK). A section of the Alu domain is not visible based on the cryo-EM data and therefore marked missing. In PyMOL: set_view (-0.699150383, -0.621524274, 0.353370398, 0.497510254, -0.067985296, 0.864776254, -0.513455927, 0.780423343, 0.356752545, 0, 0, -445.966888428, 258.757995605, 294.736541748, 289.847534180, 271.441589355, 620.492187500, -20). **c**, Secondary structure model of the 7SL RNA based on the cryo-EM and icSHAPE data (extracted from Lu et al. 2016 Cell, PMID: 27180905). The 5 SHARC-exo crosslinking sites are marked by black lines. t1 and t2 are tertiary contacts in the cryo-EM model. **d**, SHARC-exo data supporting the spatial proximities. Top panel shows the secondary structure, where the Alu and S domains are color coded. A total of 5 DGs were identified with at least 5 reads in each DG. **e**, Numbers of reads, sequence coordinates, spatial distances and helices for the 5 DGs. The one on the bottom (213-248) likely represents an alternative conformation of the helix 8. **f**, Mapping the SHARC-exo derived crosslinking sites onto the cryo-EM structure of 7SL. Color blue to red, from the 5' end to the 3' end. The nucleotide 67 is missing from the cryo-EM, therefore, the distance is a rough estimate. Nucleotides 101 and 251 are right next to each other, and overlapped in this view. **g**, A model of the alternative conformations in 7SL S domain (Redrawn from Kuglstatter et al. 2002). The folded conformation is necessary for SRP19 binding, which then recruits SRP54. Unfolding of the packed helices 6 and 8 would allow helix 8 to bend backward to make contact with 5e. This alternative open conformation was previously suggested as an assembly precursor of the SRP complex (Kuglstatter et al. 2002, PMID: 12244299). Source data are provided as a Source Data file.

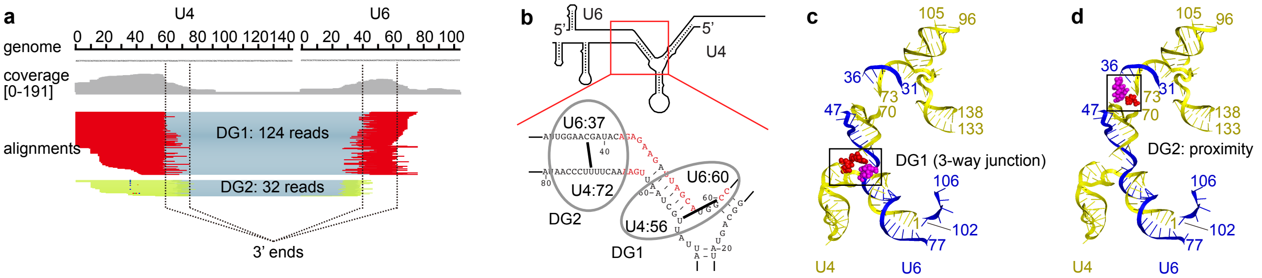

Supplementary Figure 9. SHARC-exo captures intermolecular interactions in the spliceosomal RNAs. a, SHARC-exo data for U4-U6 interactions. The numbers of reads and 3' ends of the two DGs are labeled. **b**, Secondary structure model of the U4-U6 dimer (redrawn from Patel and Steitz 2003, PMID: 14685174). SHARC-exo crosslinked sites are labeled. **c-d**, Physical locations of SHARC-exo crosslinking sites on the U5.U4/U6 tri-snRNP cryo-EM structure model (PDB:6QW6). The crosslinked sites of DG2 were missed in cryo-EM structure, but captured by SHARC-exo (U4:72 vs. U6:37). The diagram in panel d shows estimated locations. Source data are provided as a Source Data file.

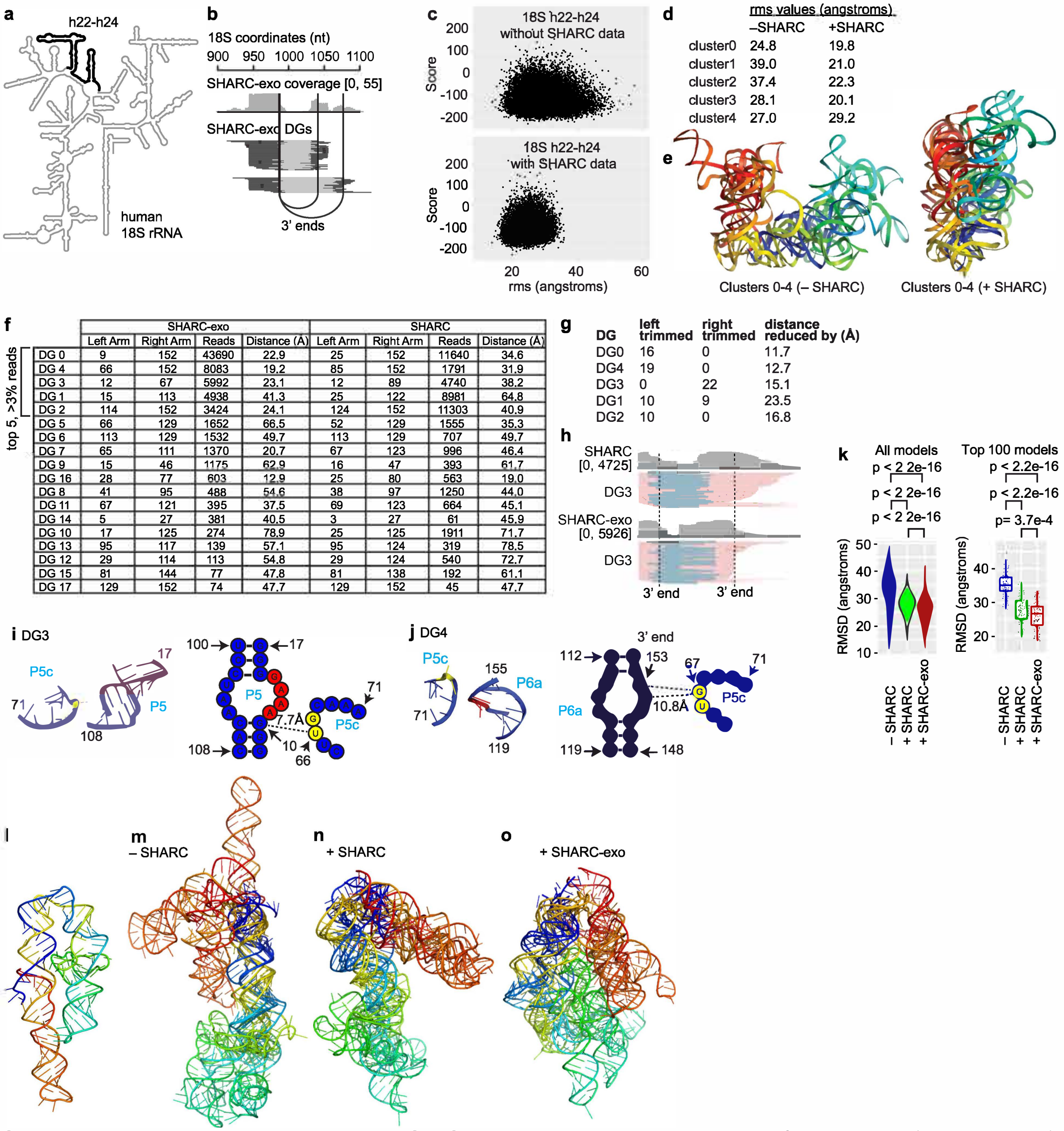

d

	rms values (angstroms)	
	–SHARC	+SHARC
cluster0	24.8	19.8
cluster1	39.0	21.0
cluster2	37.4	22.3
cluster3	28.1	20.1
cluster4	27.0	29.2

f

		SHARC-exo				SHARC			
		Left Arm	Right Arm	Reads	Distance (Å)	Left Arm	Right Arm	Reads	Distance (Å)
top 5, >3% reads	DG 0	9	152	43690	22.9	25	152	11640	34.6
	DG 4	66	152	8083	19.2	85	152	1791	31.9
	DG 3	12	67	5992	23.1	12	89	4740	38.2
	DG 1	15	113	4938	41.3	25	122	8981	64.8
	DG 2	114	152	3424	24.1	124	152	11303	40.9
	DG 5	66	129	1652	66.5	52	129	1555	35.3
	DG 6	113	129	1532	49.7	113	129	707	49.7
	DG 7	65	111	1370	20.7	67	123	996	46.4
	DG 9	15	46	1175	62.9	16	47	393	61.7
	DG 16	28	77	603	12.9	25	80	563	19.0
	DG 8	41	95	488	54.6	38	97	1250	44.0
	DG 11	67	121	395	37.5	69	123	664	45.1
	DG 14	5	27	381	40.5	3	27	61	45.9
	DG 10	17	125	274	78.9	25	125	1911	71.7
	DG 13	95	117	139	57.1	95	124	319	78.5
	DG 12	29	114	113	54.8	29	124	540	72.7
	DG 15	81	144	77	47.8	81	138	192	61.1
	DG 17	129	152	74	47.7	129	152	45	47.7

g

DG	left trimmed	right trimmed	distance reduced by (Å)
DG0	16	0	11.7
DG4	19	0	12.7
DG3	0	22	15.1
DG1	10	9	23.5
DG2	10	0	16.8

Supplementary Figure 10. Using spatial distances captured by SHARC-exo to improve Rosetta 3D modeling. a, Location of the helices h22-h24 (nucleotides 920-1090) in the human 18S rRNA. **b**, SHARC-exo data (5mM SHARC, 12h RNase R), showing DGs that captured two pairs of nucleotides in spatial proximity. Vertical lines indicate the 3' ends of the trimmed reads. **c**, Scatter plots of Rosetta rms (root-mean-square deviation) values vs. scores for the 18S h22-h24 region with or without SHARC-exo constraints. The two constraints removed the vast majority of high rms models (>35 angstroms). **d**, rms values of clusters 0-5 of the 18S h22-h24 segment with or without SHARC-exo constraints. **e**, Alignment of center models for the top 5 clusters (0-4). **f**, Table detailing the number of reads and indicated crosslinking site for each DG for the in vitro P4-P6 SHARC-exo data. DGs with reads more than 3% of the SHARC-exo library are selected for modeling analysis resulting in 5 DGs that account for 89% of SHARC-exo reads and 81% of SHARC reads. **g**, Numbers of nucleotides trimmed from the left and right arms after RNase R treatment (SHARC-exo vs. SHARC), and the improvement of distance measurements (column: distanced reduced by (Å)). **h**, Gapped reads for SHARC (top) and SHARC-exo (bottom) in DG3 (coverage listed in brackets). The vertical lines show the 3' ends (medians) under the SHARC-exo condition. **i-j**, Shortest distances between the two arms (measured on O2' atoms) in DGs 3 and 4. These distances are shorter than the distances measured at the 5th nucleotide from the 3' end, as shown in panel **f**. Un-paired residues most likely to be involved in the crosslinking are depicted in red and yellow. **k**, Violin plot of overall models RMSD as well as boxplot of the top 100 models for each modeling condition (using top 5 DGs from the SHARC and SHARC-exo conditions). Total numbers of models generated by Rosetta are as follows. –SHARC: 13132, +SHARC: 11925, +SHARC-exo: 12651. The –SHARC condition was run without tertiary contact constraints. For boxplots the median is marked by the solid line in the center of box the verticle length of the box represents the interquartile range (IQR) upper fence: 75th percentile + 1.5*IQR, lower fence: 25th percentile - 1.5*IQR, p values for Wilcox rank sum tests are shown above. **l**, Crystal structure of the P4-P6 RNA (PDB: 1HR2). **m-o**, Overlap of the top 100 models as 5 clusters, for –SHARC (m), +SHARC (n), and +SHARC-exo (o) conditions. Source data are provided as a Source Data file.

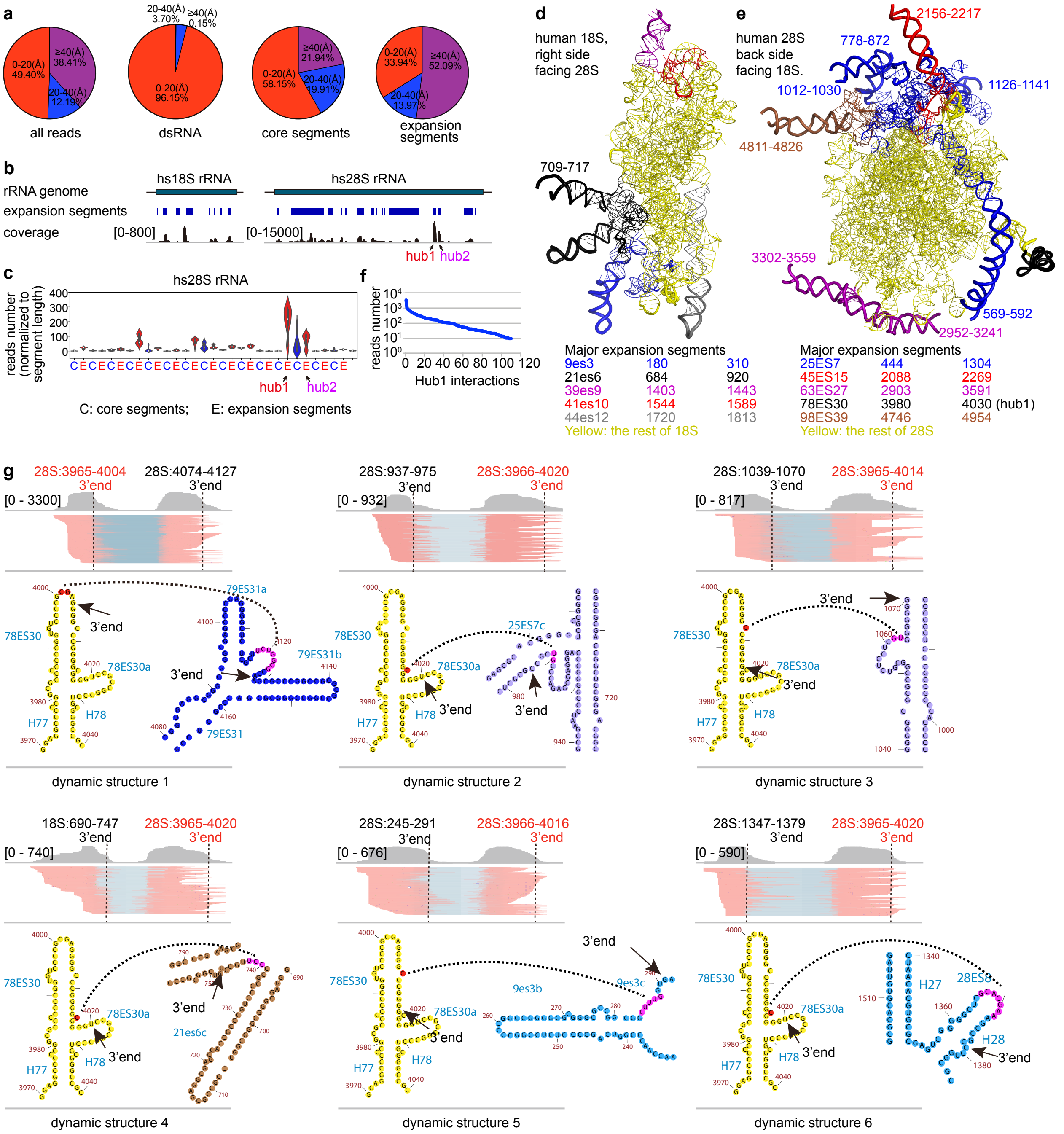

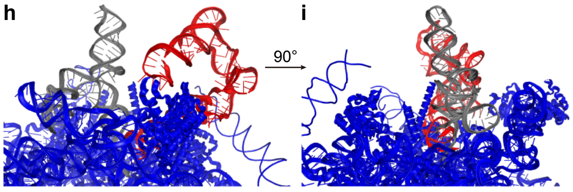

Supplementary Figure 11. SHARC-exo captures dynamic structures in the human ribosome. a, Percentages of reads with between-arm distances in three ranges: 0-20, 20-40 and ≥40Å. For crosslinks constrained by dsRNAs, the vast majority of distances (96.15%) are within 20Å. For tertiary contacts in the core and expansion segments, more distances are >20 or >40 Å. **b**, Genome coverage track showing that SHARC-exo reads with larger distance between two arms (≥40Å) are primarily mapped to18S and 28S rRNA expansion segments. The scales are different between 18S and 28S. **c**, Violin plot of per-nt reads coverage along the 28S, in core and expansion segment intervals. **d-e**, Locations of the major expansion segments on the ribosome cryo-EM structures (only expansion segments >50nts are shown). Thin lines with bases represent well positioned regions in the cryo-EM structure, while thick lines without bases represent high B values, i.e. flexible regions. Missing segments are listed next to the break points. Some of the expansion segments, even though are well positioned in this model, may be more flexible in cells, e.g. the roots of 21ES6, 44es12, and 63ES27. For example, 63ES27 missed two regions 2952-3241 and 3302-3559, which add up to 548 nucleotides, the lost of all the missed expansion segments. **f**, Numbers of reads in the hub1-interacting DGs, ranked by coverage. **g**, Details of all the 6 DGs supporting dynamic conformations between 78ES30 and its targets. **h-i**, Model of the alternative interaction between ES30 and ES31, illustrating the lack of clashes with the surface of the ribosome. Two views rotated horizontally by 90° are shown. Red: H76-H78 and ES30. Gray: H79 and ES31. Source data are provided as a Source Data file.

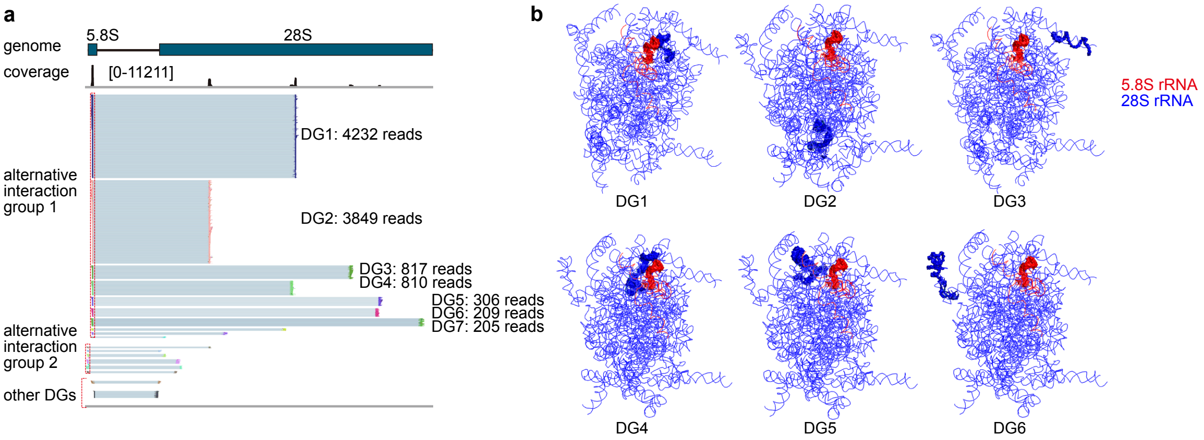

Supplementary Figure 12. SHARC-exo captures intermolecular interactions between 5.8S and 28S rRNAs. a, IGV plot showing the interactions between 5.8S and 28S rRNA. Only the duplex groups (DGs) with more than 10 reads were showed here. **b**, Physical locations of top 6 5.8S-28S rRNA interactions on the ribosome cryo-EM structure (PDB: 4V6X). Interacting regions are shown in spheres while the rest are in lines. The part of 5.8S involved in all the alternative contacts is exposed to the surface of the ribosome, making it possible to reach distant 28S helices. Source data are provided as a Source Data file.

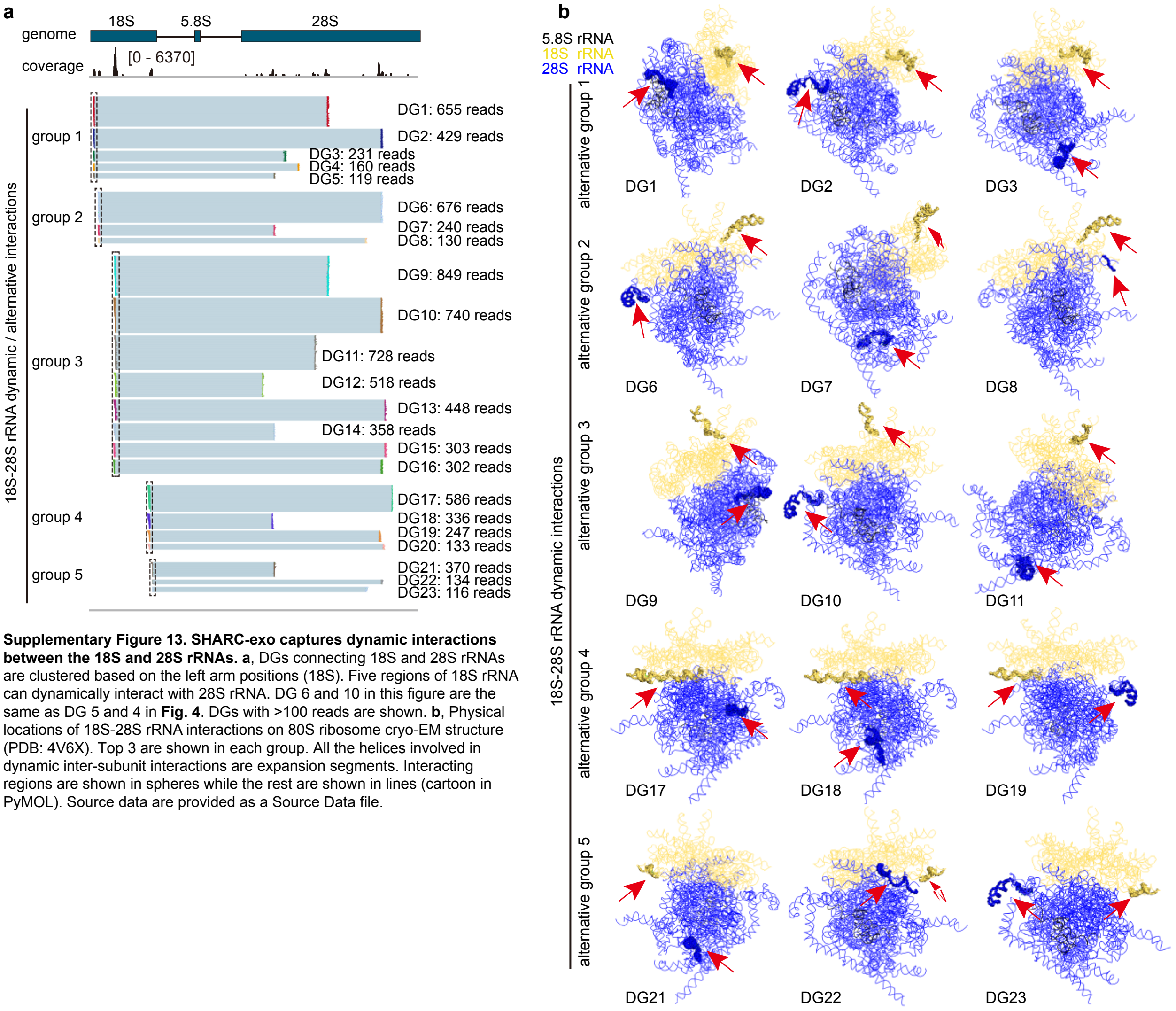

Supplementary Figure 13. SHARC-exo captures dynamic interactions between the 18S and 28S rRNAs. a, DGs connecting 18S and 28S rRNAs are clustered based on the left arm positions (18S). Five regions of 18S rRNA can dynamically interact with 28S rRNA. DG 6 and 10 in this figure are the same as DG 5 and 4 in **Fig. 4**. DGs with >100 reads are shown. **b**, Physical locations of 18S-28S rRNA interactions on 80S ribosome cryo-EM structure (PDB: 4V6X). Top 3 are shown in each group. All the helices involved in dynamic inter-subunit interactions are expansion segments. Interacting regions are shown in spheres while the rest are shown in lines (cartoon in PyMOL). Source data are provided as a Source Data file.

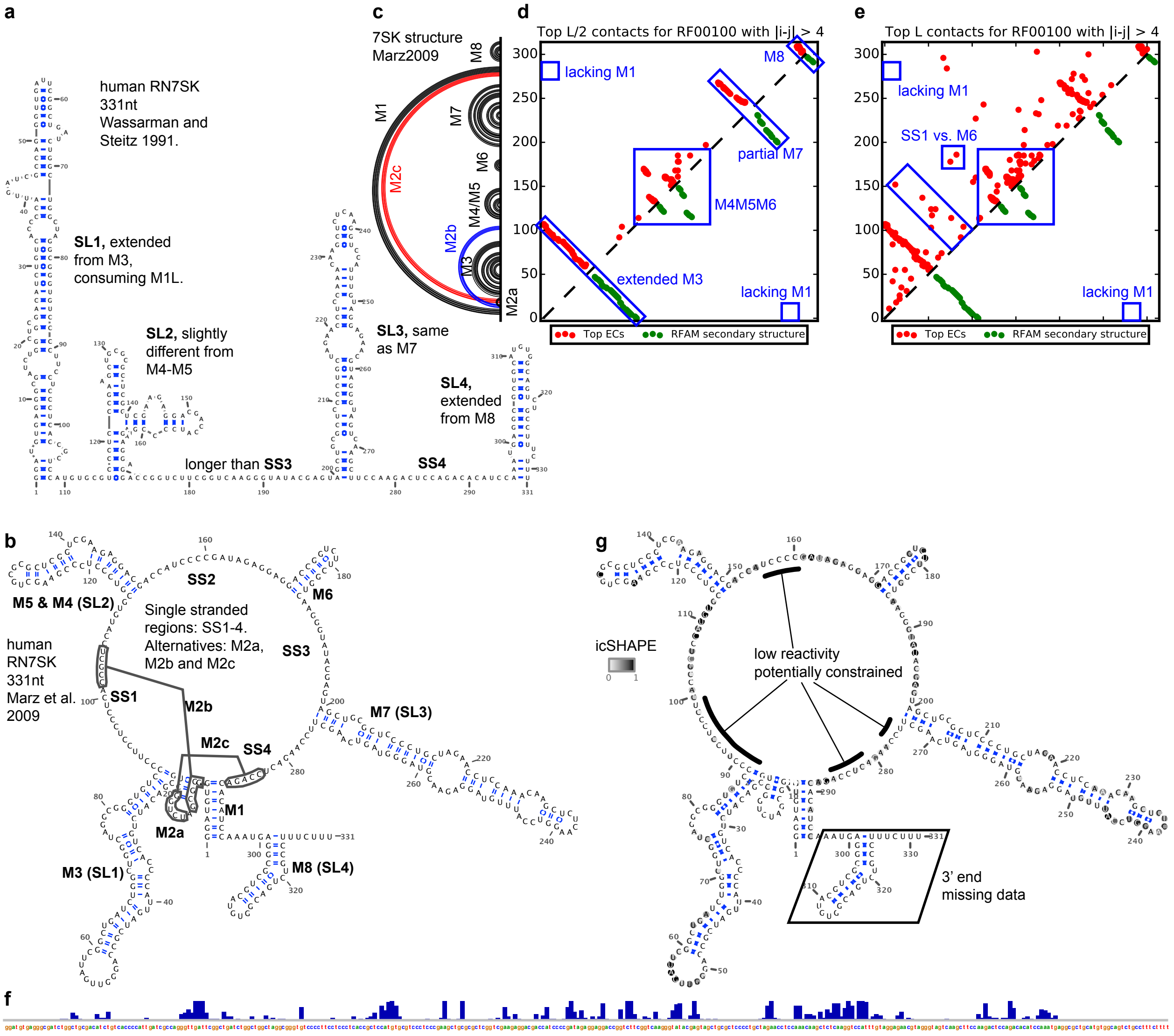

Supplementary Figure 14. icSHAPE analysis of the human 7SK RNA. a, Secondary structure model from Wassarman and Steitz 1991. SL1-4 are based on recent nomenclature in crystallographic studies, not the same as the original helices. **b**, Secondary structure model from Marz et al. 2009 (PMID: 19734296). The 8 helices are labeled M1-8, and their alternative names are indicated in the parentheses (SL1-4). The 3 M2 alternative conformations M2a-c and 4 single stranded regions SS1-4 are illustrated. **c-e,** Comparing the Marz model with (c) with EC and Rfam models (d-e). **d-e**, Contacts in the 7SK RNA identified by evolutionary coupling (EC) are shown on the top triangles (Weinreb et al. 2016. PMID: 27087444), while the Rfam model is on the bottom. The top L/2 contacts (d) are by definition more reliable than the top L contacts (e). Top L/2 contacts are almost identical with Rfam secondary structure (Rfam: RF00100), which is consistent with Marz 2009 model. However, the terminal helix M1 was not detected in either Rfam or EC. The extended M3 is also partially inconsistent with the M3 in the Marz model. **f-g**, icSHAPE data from Lu et al. 2016 (PMID: 27180905, panel c) mapped to the Marz 2009 secondary structure model (panel d). icSHAPE data were from HEK 293 cells, without any special treatment. No data were available in the first 5 and last 35 nts due to sliding window processing and primer binding. Constrained regions in the putative single-stranded regions are labeled with thick black curves. These low reactivity nucleotides are likely interacting with proteins or forming tertiary RNA structures that were not captured by phylogenetic analyses such as covariation or evolutionary coupling. However, icSHAPE alone can neither prove nor disprove long-range or tertiary structures. Source data are provided as a Source Data file.

a

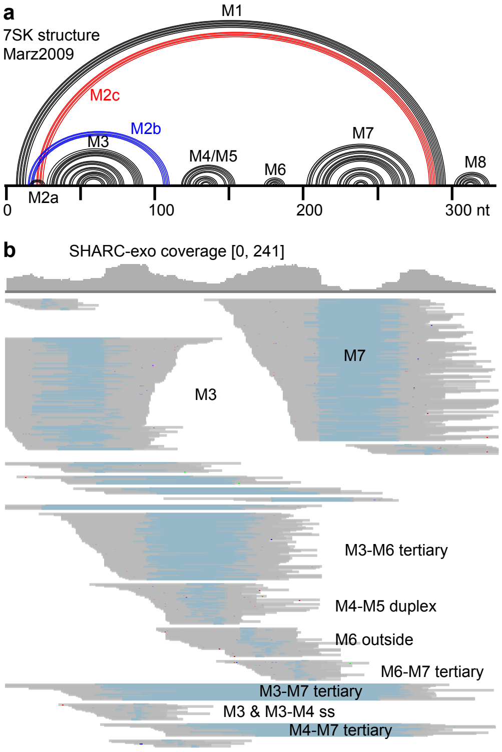

c

DG	Reads	L5	L3	R5	R3
0	156	162	211	254	290
1	123	1	24	66	97
2	73	49	97	155	186
3	44	89	124	137	159
4	31	109	160	169	197
5	21	163	196	200	217
6	17	17	66	266	284
7	17	48	88	93	109
8	13	99	126	266	286
9	10	238	267	280	325
10	7	77	110	116	139
11	10	19	49	68	132
12	11	3	26	29	49
13	8	34	62	143	166
14	6	55	82	186	225

d

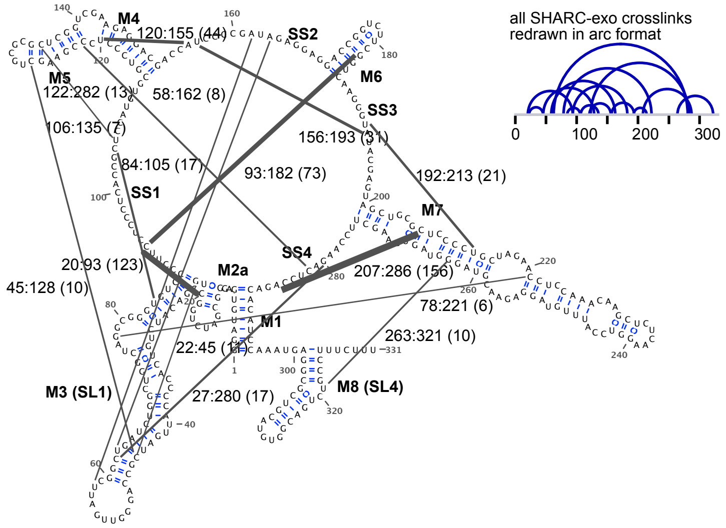

Supplementary Figure 15. SHARC-exo data for the human 7SK RNA. a, The Marz model for the 7SK RNA. Blue arcs: M2b. Red arcs: M2c. **b**, SHARC-exo reads coverage and DGs supporting the major helices and interhelical contacts. M8 was not represented in the data due to its small size and tight structure. **c**, Numbers of reads and start/end coordinates for the two arms (L5/L3 for left arm 5' and 3' ends. R5/R3 for right arm 5' and 3' ends). **d,** Mapping the crosslinks to the secondary structure model of 7SK. Same as Fig. 6a, but with more details, including the two nucleotides in contact and the numbers of reads in parentheses. Thickness of the black lines are proportionate to the square root of numbers of reads supporting each contact. An arc format is shown on the right. Source data are provided as a Source Data file.

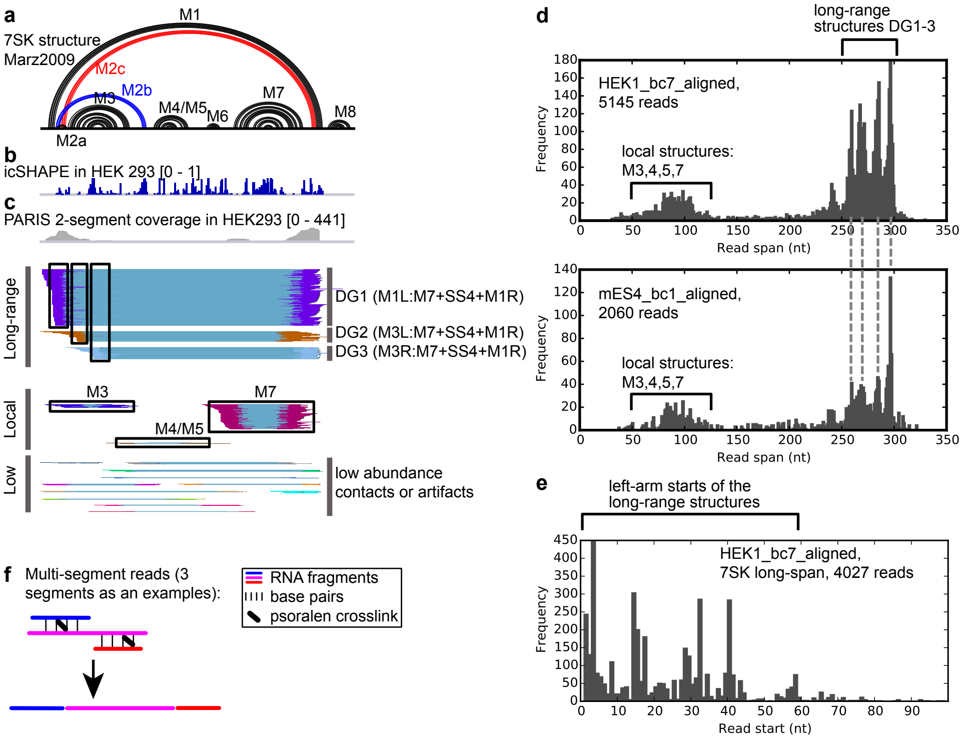

Supplementary Figure 16. PARIS analysis 7SK RNA secondary structure. a, The Marz 2009 secondary structure model for the 7SK RNA. Blue arcs: M2b. Red arcs: M2c. **b**, icSHAPE reactivity score from Lu et al. 2016. **c**, PARIS coverage and single-gap DGs clustered by CRSSANT, divided into the long-range, local and low-abundance groups. The 3 long-range DGs represent contacts between the 5' end and the 3' end. DG1 confirms the existence of M1, and the high reads coverage suggest that it is highly abundant, if not the only conformation. DGs 2 and 3 are not consistent with any secondary structures in the Wassarman and Steitz 1991 model, Marz 2009 model, EC model (Weinreb 2016), or the Rfam model, suggesting that they came from previously unknown crosslinkable contacts. The PARIS data here lacked the nucleotide resolution in the SHARC-exo, making it difficult to detect the exact crosslinked nucleotides. M6 and M8 were missed, probably due to lack of the preferred psoralen crosslinking sites (staggered uridines). The low abundance local duplexes were likely from dynamic intermediates of 7SK folding or technical artifacts of proximity ligation. **d**, Analysis of the span of all PARIS gapped reads in human and mouse cells (HEK293 and mouse ES (mES) cells, Lu et al. 2016, PMID: 27180905). There are two types of reads that correspond to local (M3,4,5 and 7) and long-range structrues. In particular, the long-range structures are further sub-divided into 4 groups, roughly corresponding to the 3 DGs. The DGs are defined by overlap on the two arms, and therefore do not correspond exactly with the groups shown here based on the read span. **e,** Plotting the start position of the long-range reads as a histogram, showing several peaks that roughly correspond to the DGs 1-3. These reads start at several distinct locations between 0-60, but also end at the same region, M7+SS4+M1R (see panel c). **f**, Diagram of multi-segment reads. Stronger crosslinking produces complexes with more than 2 RNA fragments, which can be ligated together and sequenced. Such reads indicate that these fragments are in proximity in the same molecule and the same conformation. These multi-segment reads and the 2-segment long-range structures (DG1-3) support interhelical packing of the 7SK RNA. Source data are provided as a Source Data file.

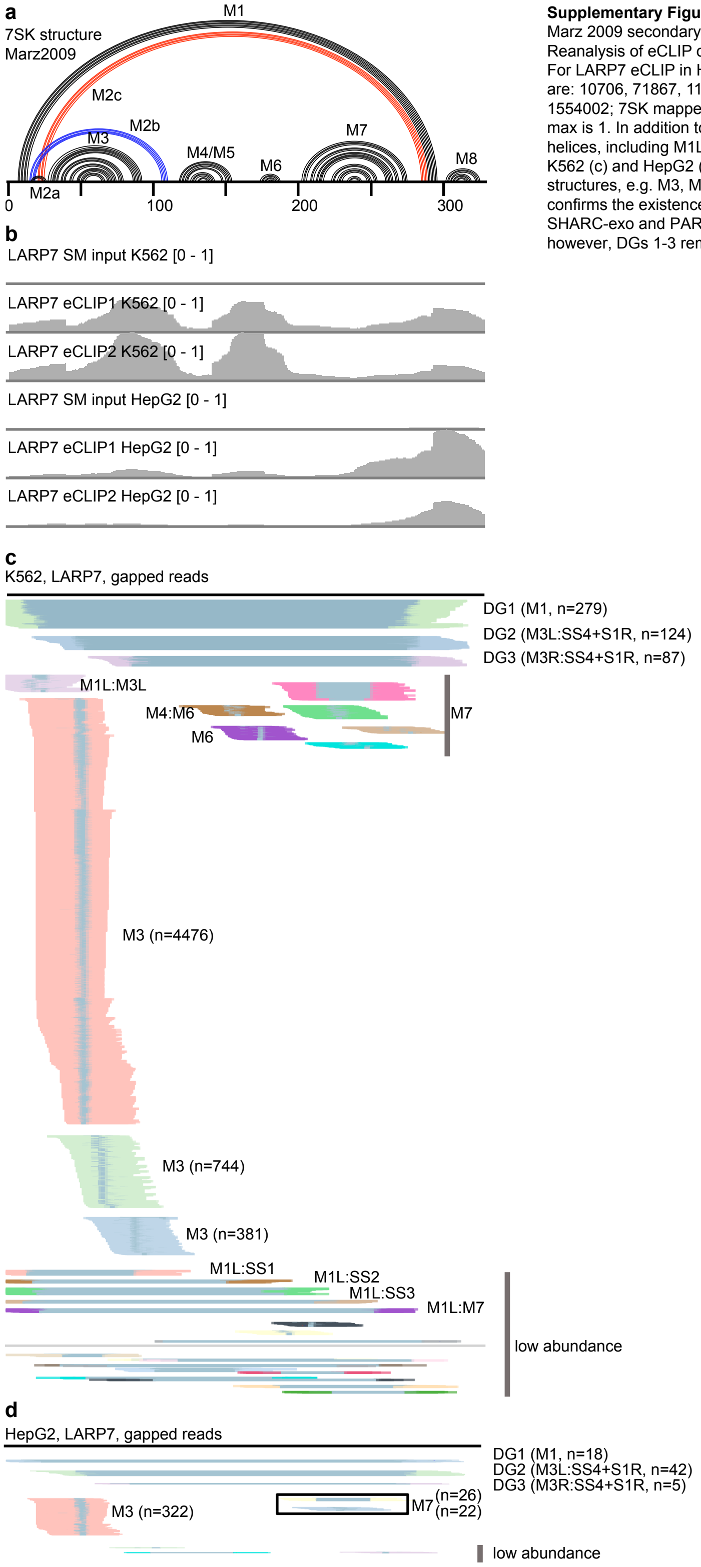

Supplementary Figure 17. LARP7 eCLIP reveals long-range contacts in the 7SK RNA. a, The Marz 2009 secondary structure model for the 7SK RNA. Blue arcs: M2b. Red arcs: M2c. **b**, Reanalysis of eCLIP data from K562 and HepG2 cells (Van Nostrand et al. 2016, PMID: 27018577). For LARP7 eCLIP in HepG2, total mapped reads are 5674934, 1381014, 446830; 7SK mapped reads are: 10706, 71867, 11047. For LARP7 eCLIP in K562, total mapped reads are 7302572, 1790120, 1554002; 7SK mapped reads are: 50306, 517215, 673789. The samples were normalized so that the max is 1. In addition to the primary binding site on the 3' end M8 region, LARP7 also binds the 5' end helices, including M1L, M2, M3, and between M4/M5/M6. **c-d**, Gapped reads from LARP7 eCLIP in K562 (c) and HepG2 (d) cells were clustered into DGs using CRSSANT. In addition to capturing local structures, e.g. M3, M6, M7, these gapped reads revealed long-range structures DG1-3. DG1 again confirms the existence of M1 helix, while DGs 2-3 are consistent with newly identified contacts by SHARC-exo and PARIS. The total numbers of reads for 7SK were lower for the HepG2 cells, however, DGs 1-3 remain identifiable. Source data are provided as a Source Data file.

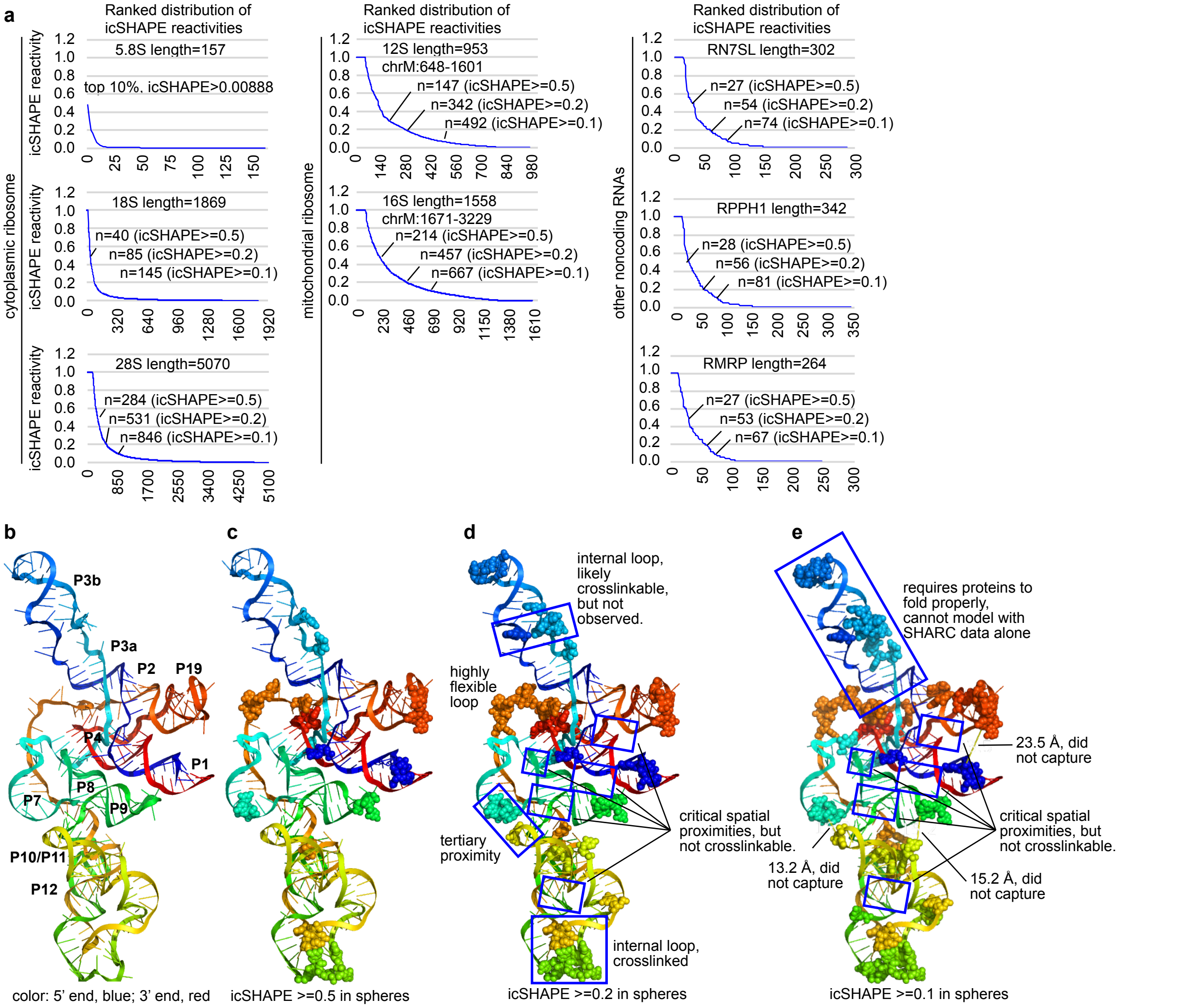

Supplementary Figure 18. Analysis of RNA icSHAPE reactivity and limitations of SHARC-exo. a, icSHAPE reactivity for abundant noncoding RNAs was extracted from our recently published sequencing data (Lu et al. 2016, PMID: 27180905). Numbers of reactive nucleotides at different cutoff levels are labeled. The 18S and 5.8S rRNAs are far less reactive compared to other RNAs. The lower reactivity of 18S compared to 28S is probably due to the lower fraction of expansion segments. Typically <10% nucleotides have >0.5 SHAPE reactivity in each RNA. The higher reactivity of the mitochondrial ribosome is likely due to its smaller size and more primitive form. which allows SHAPE reagent to access. These distributions both confirmed the applicability of SHARC-exo to various RNAs, and also showed the limitations. **b-e,** Mapping of icSHAPE reactive nucleotides onto the RNase P RNA (RPPH1) cryo-EM structure model (PDB: 6AHR). RNA was colored blue to red from the 5' end to the 3' end. Even at a very low threshold, many of the critical regions remain non-crosslinkable, due to secondary structure or protein constraints (panel e). Source data are provided as a Source Data file.

Supplementary Table 1. SHARC-exo library statistics. Unique fraction: reads_unique/reads_raw. Reads_min20: reads with minimal length of 20nts. Total_min20: summing the two libraries for each condition. Min20 fraction: reads_min20/reads_unique
Gapped fraction: (gap1+gapm+chimeric+overlapping_chimeric)/input. Bad alignments represent a small fraction of all alignments, and therefore they are ignored.

		Preprocessing						Mapping								
**samples**	**barcodes**	**reads_raw**	**reads_unique**	**unique fraction**	**reads_min20**	**min20 fraction**	**total_min20**	**total**	**input**	**nongapped**	**gap1**	**gapm**	**chimeric**	**overlapping chimeric**	**bad alignments**	**gapped fraction**
HeLa5mMDPI_exo0h	R701	8.995.612	8.023.454	89,19%	5.564.497	69,35%	11.616.794	11.334.506	10.807.624	9.970.086	620.240	8.006	175.680	13.473	20.139	7,56%
HeLa5mMDPI_exo0h	R701	9809777	8703404	88,72%	6.052.297	69,54%										
HeLa5mMDPI_exo2h	R723	2.132.552	1.922.211	90,14%	739.272	38,46%	1.537.923	1.327.685	1.278.072	1.222.774	31.552	299	21.247	178	2.022	4,17%
HeLa5mMDPI_exo2h	R723	2313864	2073503	89,61%	798.651	38,52%										
HeLa5mMDPI_exo12h	R702	14.606.757	12.658.542	86,66%	8.160.003	64,46%	17.028.746	16.066.509	15.464.518	14.517.522	728.442	8.453	186.761	7.030	16.310	6,02%
HeLa5mMDPI_exo12h	R702	15913543	13724191	86,24%	8.868.743	64,62%										
HeLa12.5mMDPI_exo0h	R703	6.385.176	5.838.913	91,44%	4.438.825	76,02%	9.290.218	9.363.603,00	8.903.281	8.145.260	610.889	7.994	110.184	15.334	13.620	8,36%
HeLa12.5mMDPI_exo0h	R703	6992022	6367057	91,06%	4.851.393	76,20%										
HeLa12.5mMDPI_exo2h	R708	7.135.624	6.690.540	93,76%	4.583.161	68,50%	9.569.696	8.518.901	8.024.575	7.193.377	630.395	14.432	168.430	5.584	12.357	10,20%
HeLa12.5mMDPI_exo2h	R708	7779168	7270955	93,47%	4.986.535	68,58%										
HeLa12.5mMDPI_exo12h	R707	11.974.629	10.483.576	87,55%	5.523.174	52,68%	11.513.789	10.699.524	10.094.922	9.027.326	861.318	16.856	160.613	12.517	16.292	10,41%
HeLa12.5mMDPI_exo12h	R707	13039931	11351734	87,05%	5.990.615	52,77%										
HeLa12.5mMDPI_exo24h	R704	10.070.228	7.198.662	71,48%	814.144	11,31%	1.658.879	1.145.588	1.100.338	1.062.448	23.236	255	12.661	150	1.588	3,30%
HeLa12.5mMDPI_exo24h	R704	10912598	7659816	70,19%	844.735	11,03%										
HeLa25mMDPI_exo2h	R709	5.379.101	5.247.760	97,56%	4.282.122	81,60%	8.953.498	9.045.708	8.332.858	7.103.605	993.214	16.793	181.188	16.009	22.049	14,49%
HeLa25mMDPI_exo2h	R709	5875549	5723068	97,40%	4.671.376	81,62%										
HeLa25mMDPI_exo24h	R717	5.102.976	4.714.432	92,39%	2.951.322	62,60%	6.158.342	5.777.797	5.468.182	4.994.332	354.226	4.906	94.987	7.082	12.649	8,43%
HeLa25mMDPI_exo24h	R717	5565513	5121082	92,01%	3.207.020	62,62%										

Supplementary References

1 Zhang, M. *et al.* Optimized photochemistry enables efficient analysis of dynamic RNA structuromes and interactomes in genetic and infectious diseases. *Nat Commun* **12**, 2344, doi:10.1038/s41467-021-22552-y (2021).
2 Lu, Z., Gong, J. & Zhang, Q. C. PARIS: Psoralen Analysis of RNA Interactions and Structures with High Throughput and Resolution. *Methods Mol Biol* **1649**, 59-84, doi:10.1007/978-1-4939-7213-5_4 (2018).
3 Flynn, R. A. *et al.* Transcriptome-wide interrogation of RNA secondary structure in living cells with icSHAPE. *Nat Protoc* **11**, 273-290, doi:10.1038/nprot.2016.011 (2016).
4 Dobin, A. *et al.* STAR: ultrafast universal RNA-seq aligner. *Bioinformatics* **29**, 15-21, doi:10.1093/bioinformatics/bts635 (2013).
5 Fischer-Hwang, I., Lu, Z., Zou, J. & Weissman, T. Vol. https://www.biorxiv.org/content/10.1101/668491v1 (Biorxiv, 2019).
6 Robinson, J. T. *et al.* Integrative genomics viewer. *Nat Biotechnol* **29**, 24-26, doi:10.1038/nbt.1754 (2011).
7 Lu, X. J., Bussemaker, H. J. & Olson, W. K. DSSR: an integrated software tool for dissecting the spatial structure of RNA. *Nucleic Acids Res* **43**, e142, doi:10.1093/nar/gkv716 (2015).
8 Anger, A. M. *et al.* Structures of the human and Drosophila 80S ribosome. *Nature* **497**, 80-85, doi:10.1038/nature12104 (2013).
9 Chandramouli, P. *et al.* Structure of the mammalian 80S ribosome at 8.7 A resolution. *Structure* **16**, 535-548, doi:10.1016/j.str.2008.01.007 (2008).
10 Wakeman, J. A. & Maden, B. E. 28 S ribosomal RNA in vertebrates. Locations of large-scale features revealed by electron microscopy in relation to other features of the sequences. *Biochem J* **258**, 49-56, doi:10.1042/bj2580049 (1989).
11 Cheng, C. Y. *et al.* Consistent global structures of complex RNA states through multidimensional chemical mapping. *Elife* **4**, e07600, doi:10.7554/eLife.07600 (2015).
12 *The hub for Rosetta modeling software*, <https://www.rosettacommons.org/> (2020).
13 Lu, Z. *et al.* RNA Duplex Map in Living Cells Reveals Higher-Order Transcriptome Structure. *Cell* **165**, 1267-1279, doi:10.1016/j.cell.2016.04.028 (2016).
14 Van Nostrand, E. L. *et al.* Robust transcriptome-wide discovery of RNA-binding protein binding sites with enhanced CLIP (eCLIP). *Nature methods* **13**, 508-514, doi:10.1038/nmeth.3810 (2016).
