# Supplementary material for: Chemical reversible crosslinking enables measurement of RNA 3D distances and alternative conformations in cells
Source: Nat Commun. 2022 Feb 17;13:911. doi: 10.1038/s41467-022-28602-3 (PMC8854666; doi:10.1038/s41467-022-28602-3)
Supplement: Supplementary file 2 — Reporting Summary [file 41467_2022_28602_MOESM2_ESM.pdf]

nature portfolio

Corresponding author(s): Zhipeng Lu
Willem Velema

Last updated by author(s): Dec, 09,2021

Reporting Summary

Nature Portfolio wishes to improve the reproducibility of the work that we publish. This form provides structure for consistency and transparency in reporting. For further information on Nature Portfolio policies, see our Editorial Policies and the Editorial Policy Checklist.

Statistics

For all statistical analyses, confirm that the following items are present in the figure legend, table legend, main text, or Methods section.

n/a	Confirmed	
☐	☒	The exact sample size (*n*) for each experimental group/condition, given as a discrete number and unit of measurement
☐	☒	A statement on whether measurements were taken from distinct samples or whether the same sample was measured repeatedly
☐	☒	The statistical test(s) used AND whether they are one- or two-sided<br>*Only common tests should be described solely by name; describe more complex techniques in the Methods section.*
☐	☒	A description of all covariates tested
☐	☒	A description of any assumptions or corrections, such as tests of normality and adjustment for multiple comparisons
☐	☒	A full description of the statistical parameters including central tendency (e.g. means) or other basic estimates (e.g. regression coefficient) AND variation (e.g. standard deviation) or associated estimates of uncertainty (e.g. confidence intervals)
☐	☒	For null hypothesis testing, the test statistic (e.g. *F*, *t*, *r*) with confidence intervals, effect sizes, degrees of freedom and *P* value noted<br>*Give P values as exact values whenever suitable.*
☒	☐	For Bayesian analysis, information on the choice of priors and Markov chain Monte Carlo settings
☐	☒	For hierarchical and complex designs, identification of the appropriate level for tests and full reporting of outcomes
☐	☒	Estimates of effect sizes (e.g. Cohen's *d*, Pearson's *r*), indicating how they were calculated

Our web collection on statistics for biologists contains articles on many of the points above.

Software and code

Policy information about availability of computer code

Data collection	1. Nucleotides gels were acquired and analyzed by Bio-Rad Image System (Image Lab software, v6.0.1) and iBright FL1500 Image System (iBright Analysis Software, v3.1.2) and safeVIEW-MINI2 Imaging System.<br>2. SHARC-seq reads sequences were acquired using Illumina instrumentation and software (Miseq and NovaSeq 6000 System, bcl2fastq2 Conversion Software v2.20.0).
Data analysis	All software and code used in this study has been described in published literature (Trimmomatic v0.36, STAR v2.7.0f, SAMtools v1.8, IGV v2.8.13, DSSR v1.7.7, Awk v4.2.0, bedtools v2.29.2, Rosetta Software v2020.08.61146, Image Lab software, v6.0.1, iBright Analysis Software, v3.1.2, ImageJ software V1.52t, PyMOL system (Educational version, https://pymol.org/2/)), or are custom scripts available on GitHub (https://github.com/zhipenglu/CRSSANT and https://github.com/minjiezhang-usc/SHARC-seq).

For manuscripts utilizing custom algorithms or software that are central to the research but not yet described in published literature, software must be made available to editors and reviewers. We strongly encourage code deposition in a community repository (e.g. GitHub). See the Nature Portfolio guidelines for submitting code & software for further information.

Data

Policy information about availability of data

All manuscripts must include a data availability statement. This statement should provide the following information, where applicable:

- Accession codes, unique identifiers, or web links for publicly available datasets
- A description of any restrictions on data availability
- For clinical datasets or third party data, please ensure that the statement adheres to our policy

The raw and processed SHARC sequencing data was deposited to NCBI GEO with accession number GSE167812. All PDB data are available via Protein Data Bank

(4V6X, 1HR2, 6AHR, 6FRK, 6QW6).

Field-specific reporting

Please select the one below that is the best fit for your research. If you are not sure, read the appropriate sections before making your selection.

- [x] Life sciences
- [ ] Behavioural & social sciences
- [ ] Ecological, evolutionary & environmental sciences

For a reference copy of the document with all sections, see nature.com/documents/nr-reporting-summary-flat.pdf

Life sciences study design

All studies must disclose on these points even when the disclosure is negative.

Sample size	No sample size calculations were performed. Sample size was determined to be adequate based on the magnitude and consistency of measurable differences between groups.
Data exclusions	No data was excluded from analysis.
Replication	Each experiments was performed independently at least two times. SHARC sequencing were performed using three different concentration of DPI (5mM, 12.5mM and 25mM). All experiments were highly reproducible.
Randomization	The cells were randomly assigned to treated or no-treated for this study. For comparison of exo and non-exo treatments, crosslinked RNAs were randomly assigned to control and experimental groups.
Blinding	The investigators were not blinded during data collection since they were in vivo studies in which the treat groups needed to be clear when performing the experiments. However, SHARC-seq samples were processed by separate scientists and each data set was analyzed by separate bioinformaticians.

Reporting for specific materials, systems and methods

We require information from authors about some types of materials, experimental systems and methods used in many studies. Here, indicate whether each material, system or method listed is relevant to your study. If you are not sure if a list item applies to your research, read the appropriate section before selecting a response.

Materials & experimental systems

n/a	Involved in the study	
x		Antibodies
	x	Eukaryotic cell lines
x		Palaeontology and archaeology
x		Animals and other organisms
x		Human research participants
x		Clinical data
x		Dual use research of concern

Methods

n/a	Involved in the study	
x		ChIP-seq
x		Flow cytometry
x		MRI-based neuroimaging

Eukaryotic cell lines

Policy information about cell lines

Cell line source(s)	HEK293T (CRL-3216), HeLa (CCL-2) are purchased from ATCC.
Authentication	HEK293T and HeLa cells were frequently checked by the morphological features and the RNA expression profile, but not authenticated.
Mycoplasma contamination	No mycoplasma contamination was detected in these cells.
Commonly misidentified lines (See ICLAC register)	No commonly misidentified cell line was used.
